# Supplementary figures and images for: Obox4 promotes zygotic genome activation upon loss of Dux
Source: eLife. 2024 Jun 24;13:e95856. doi: 10.7554/eLife.95856 (PMC11196112; doi:10.7554/eLife.95856)

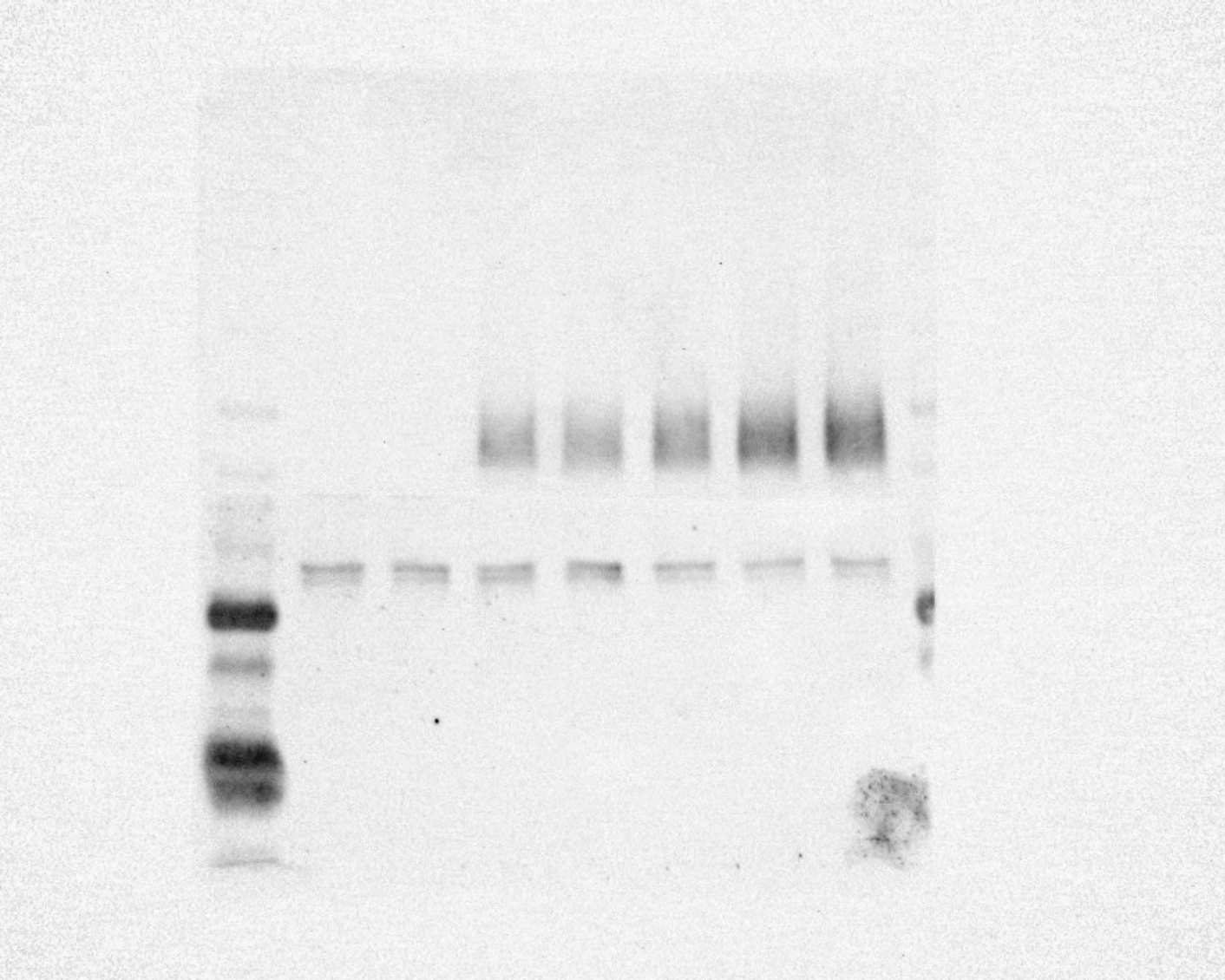

Supplement: Figure 2—source data 1. [file elife-95856-fig2-data1.zip › Figure 2 - Source Data/OBOX4_induce.tif]

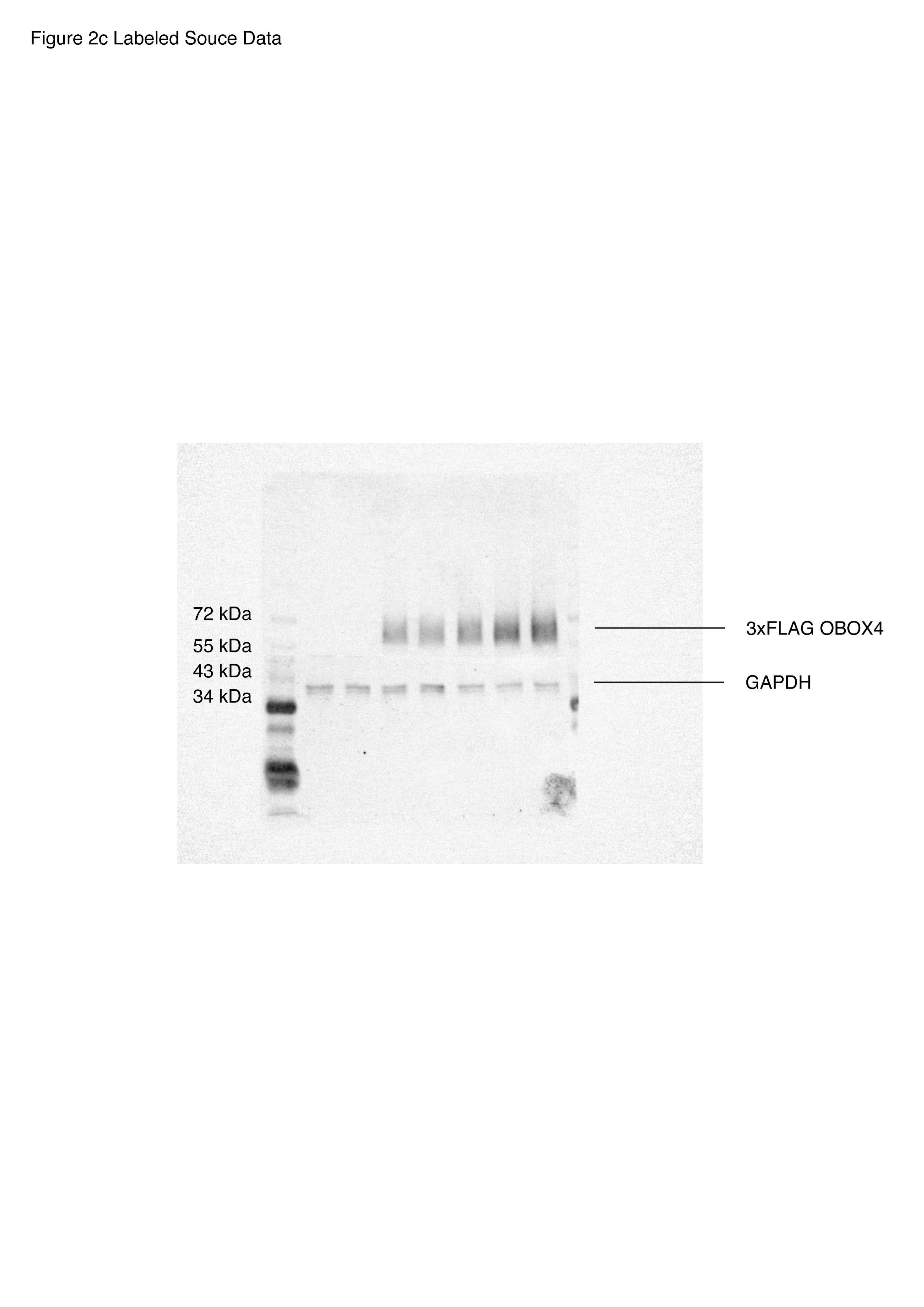

Supplement: Figure 2—source data 1. [file elife-95856-fig2-data1.zip › Figure 2 - Source Data/Figure 2 - Labeled Source Data.png]

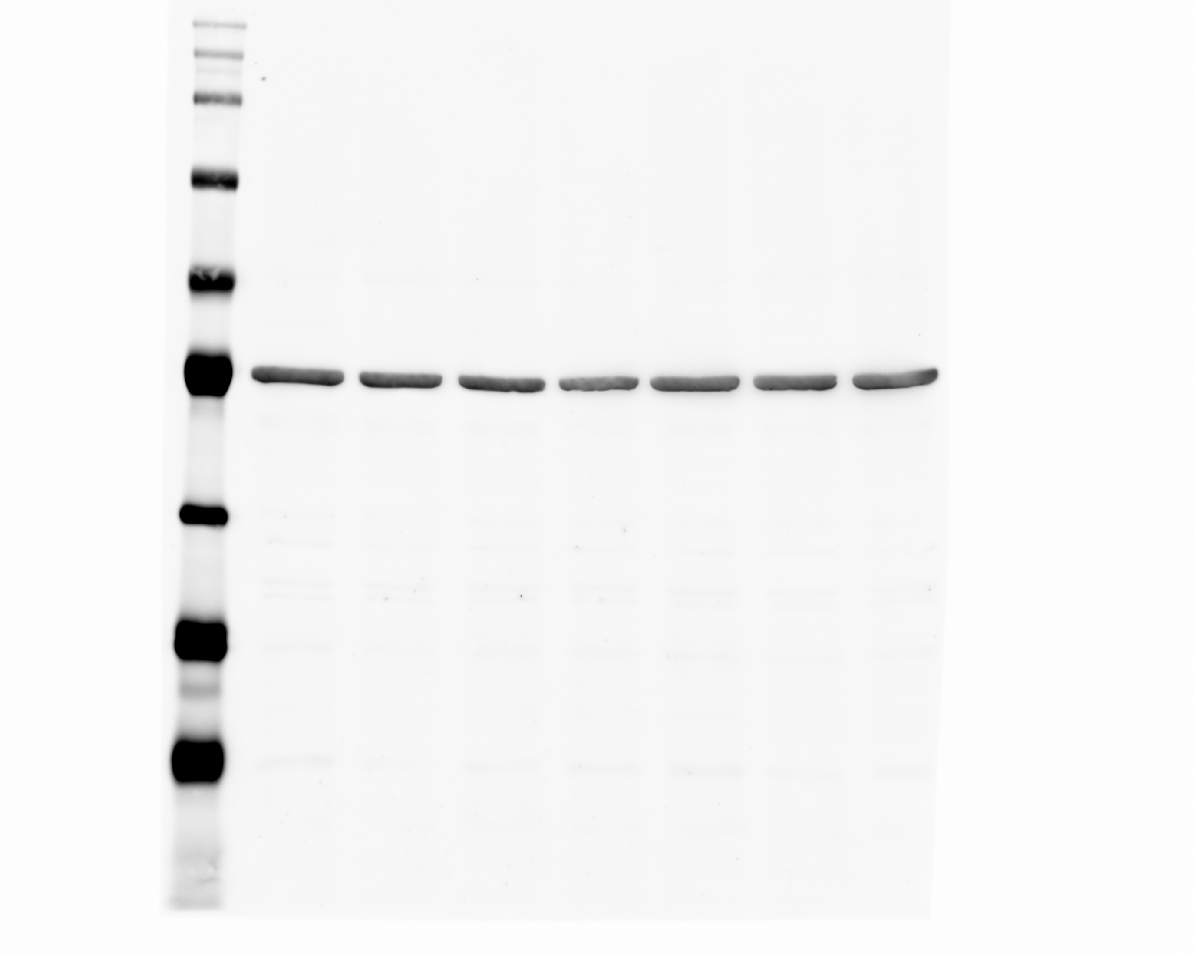

Supplement: Figure 2—figure supplement 1—source data 1. [file elife-95856-fig2-figsupp1-data1.zip › Supplementary Figure 1 - Source Data/20210702LTR_tdTomato_Homeobox_Tubb.tif]

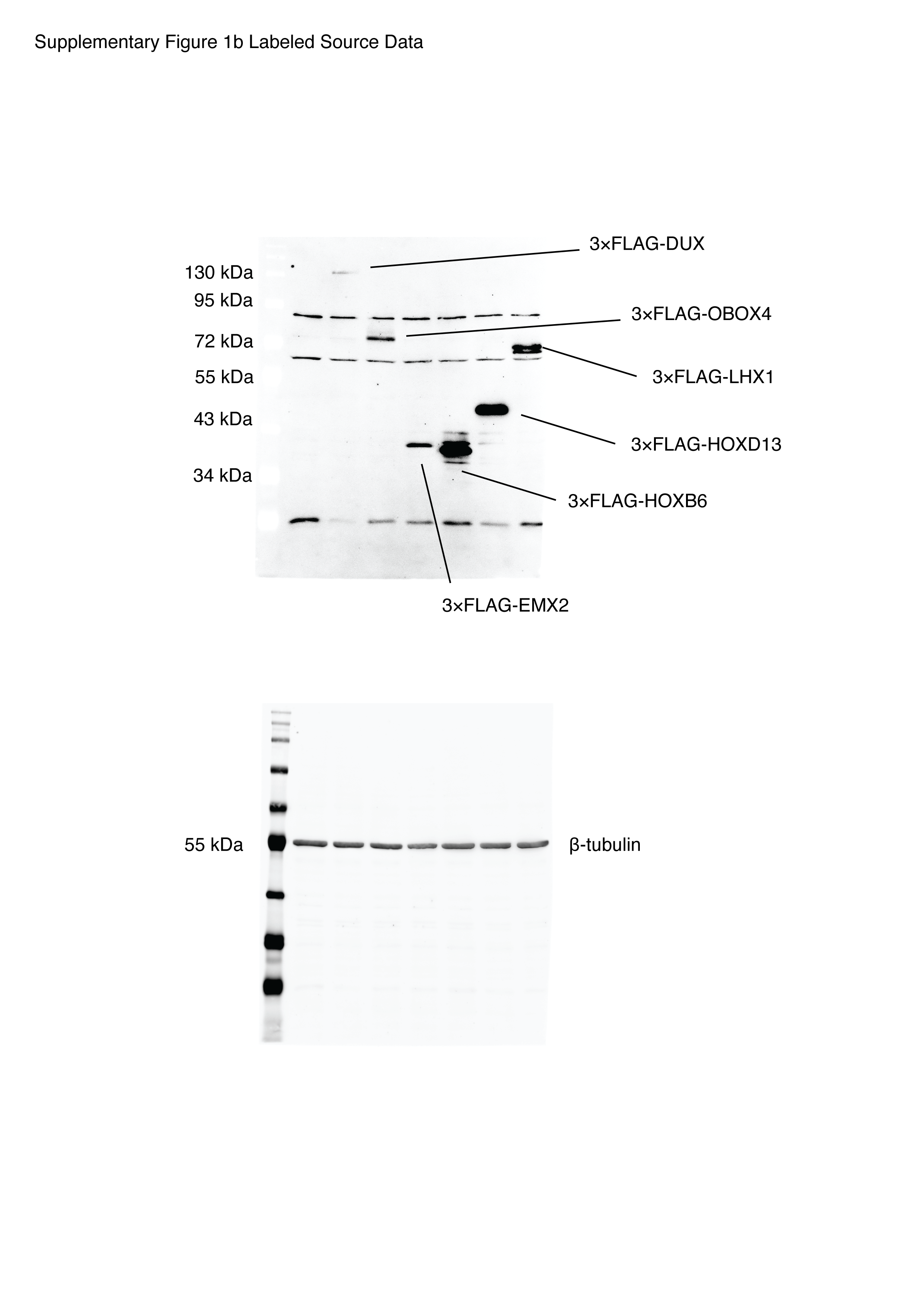

Supplement: Figure 2—figure supplement 1—source data 1. [file elife-95856-fig2-figsupp1-data1.zip › Supplementary Figure 1 - Source Data/Supplementary Figure 1b - Labeled Source Data.png]

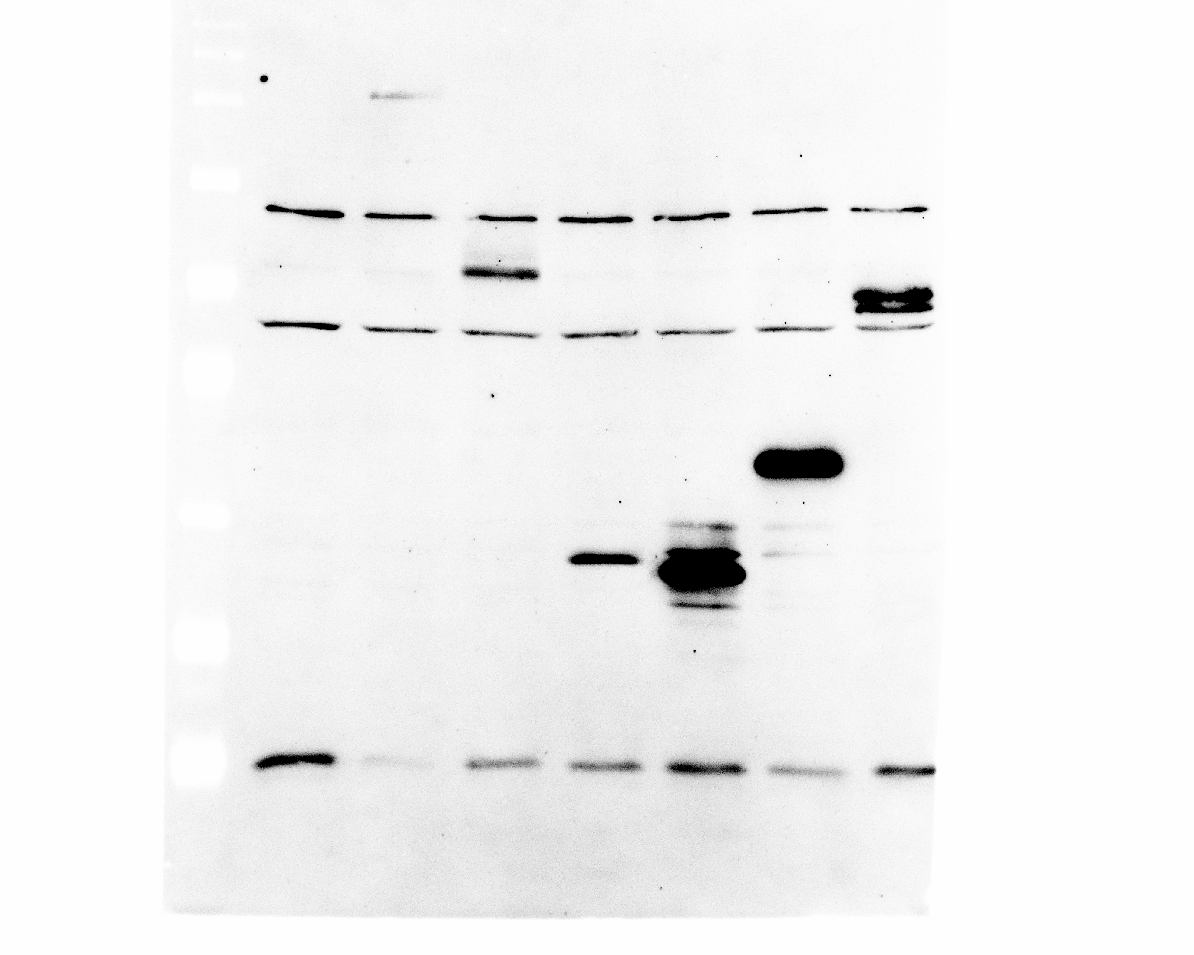

Supplement: Figure 2—figure supplement 1—source data 1. [file elife-95856-fig2-figsupp1-data1.zip › Supplementary Figure 1 - Source Data/20210702LTR_tdTomato_Homeobox_FLAG.tif]

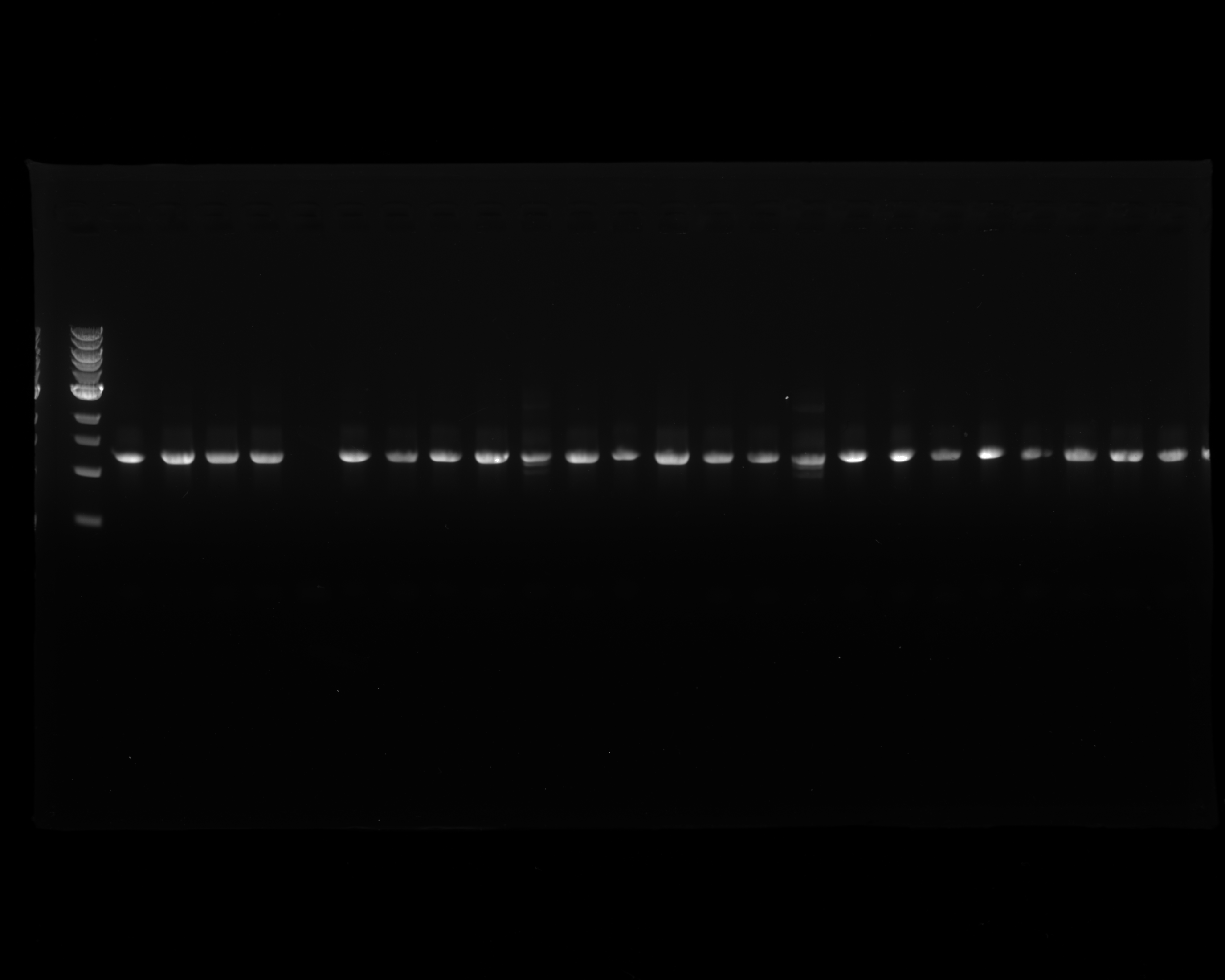

Supplement: Figure 3—figure supplement 3—source data 1. [file elife-95856-fig3-figsupp3-data1.zip › Supplementary Figure 5 - Source Data 1/mESC_Obox4_KO_27_A'+B'.tif]

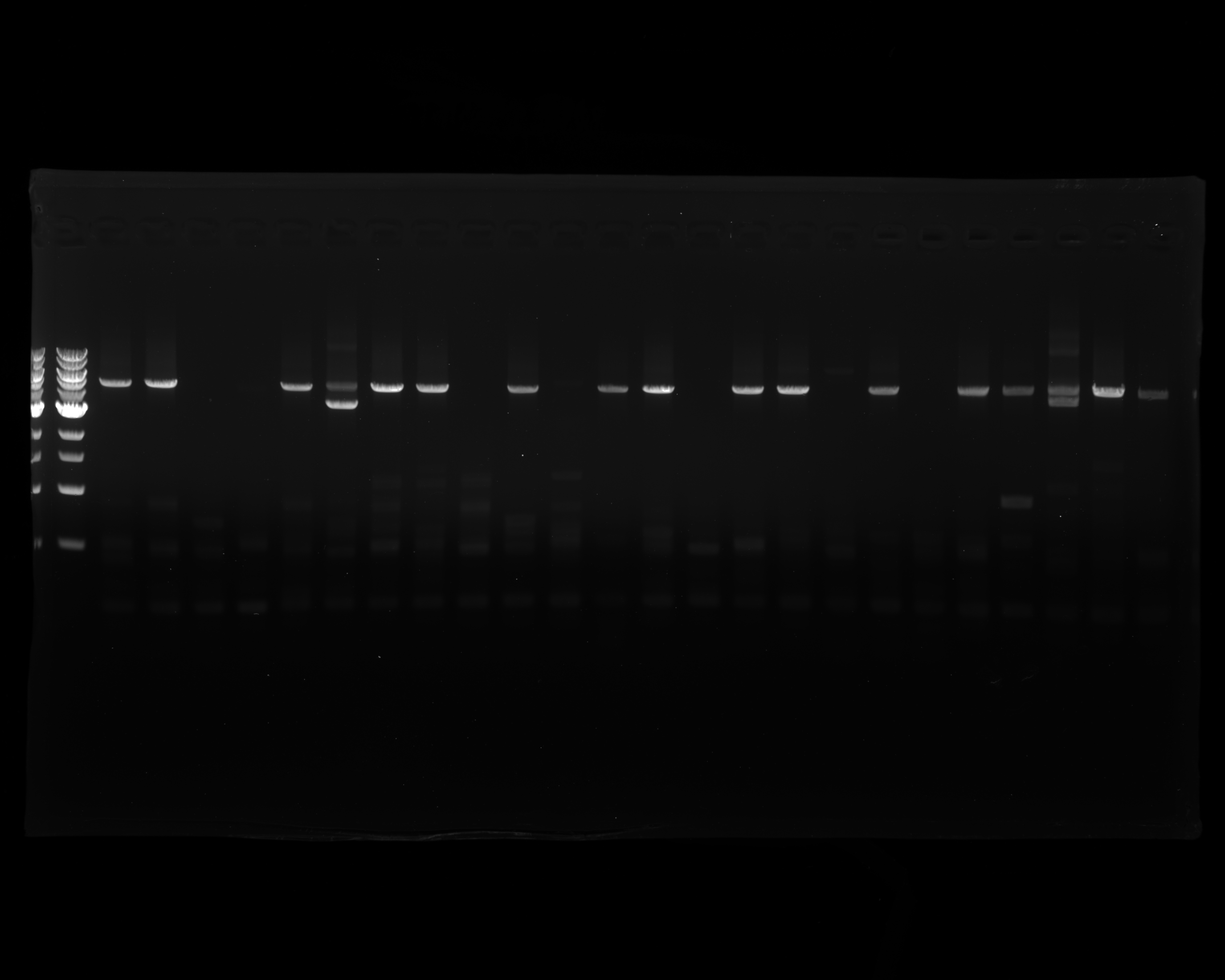

Supplement: Figure 3—figure supplement 3—source data 1. [file elife-95856-fig3-figsupp3-data1.zip › Supplementary Figure 5 - Source Data 1/mESC_Obox4_KO_27_C'+D'.tif]

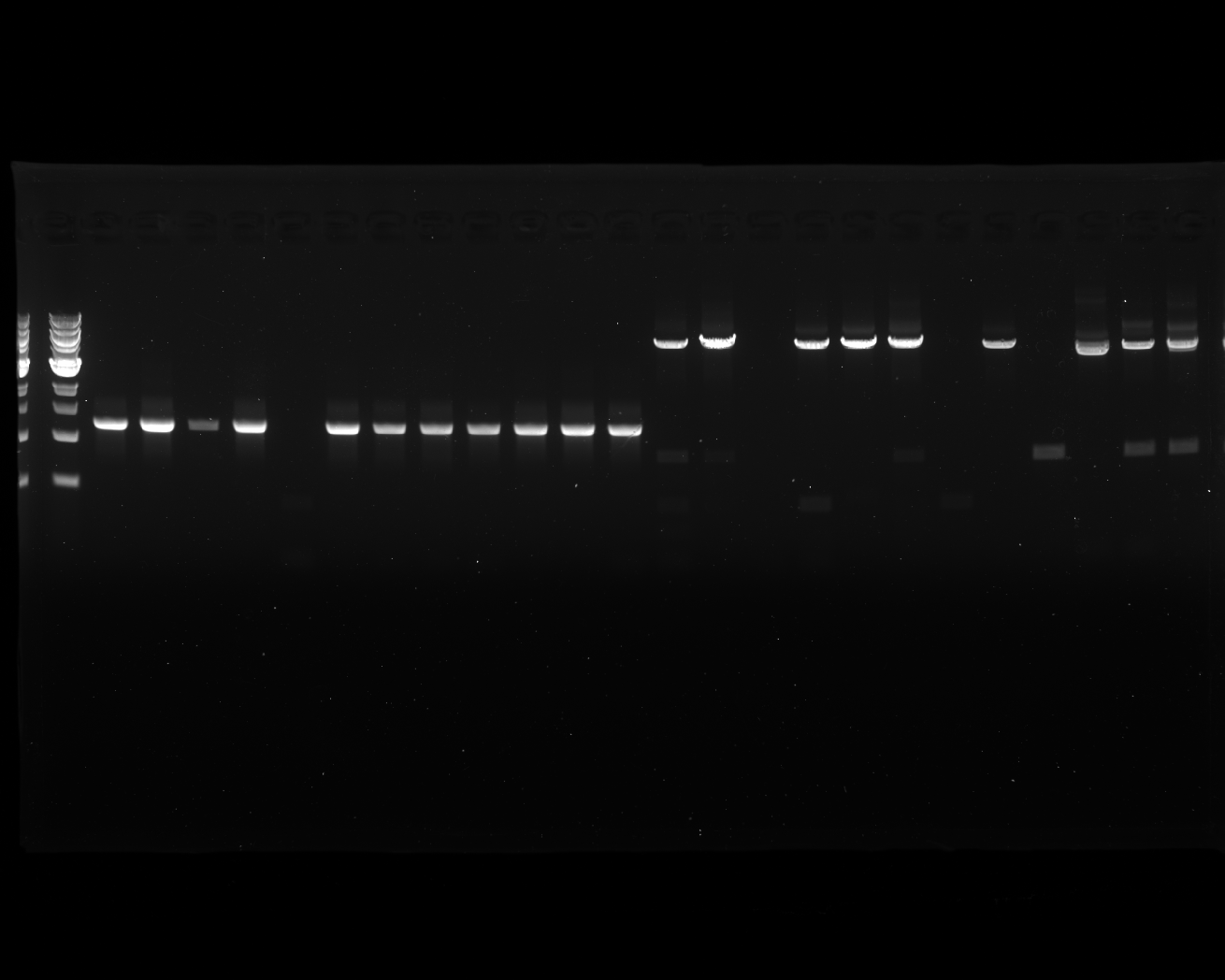

Supplement: Figure 3—figure supplement 3—source data 1. [file elife-95856-fig3-figsupp3-data1.zip › Supplementary Figure 5 - Source Data 1/mESC_Obox4_KO_413_A'+B'_A'+D'.tif]

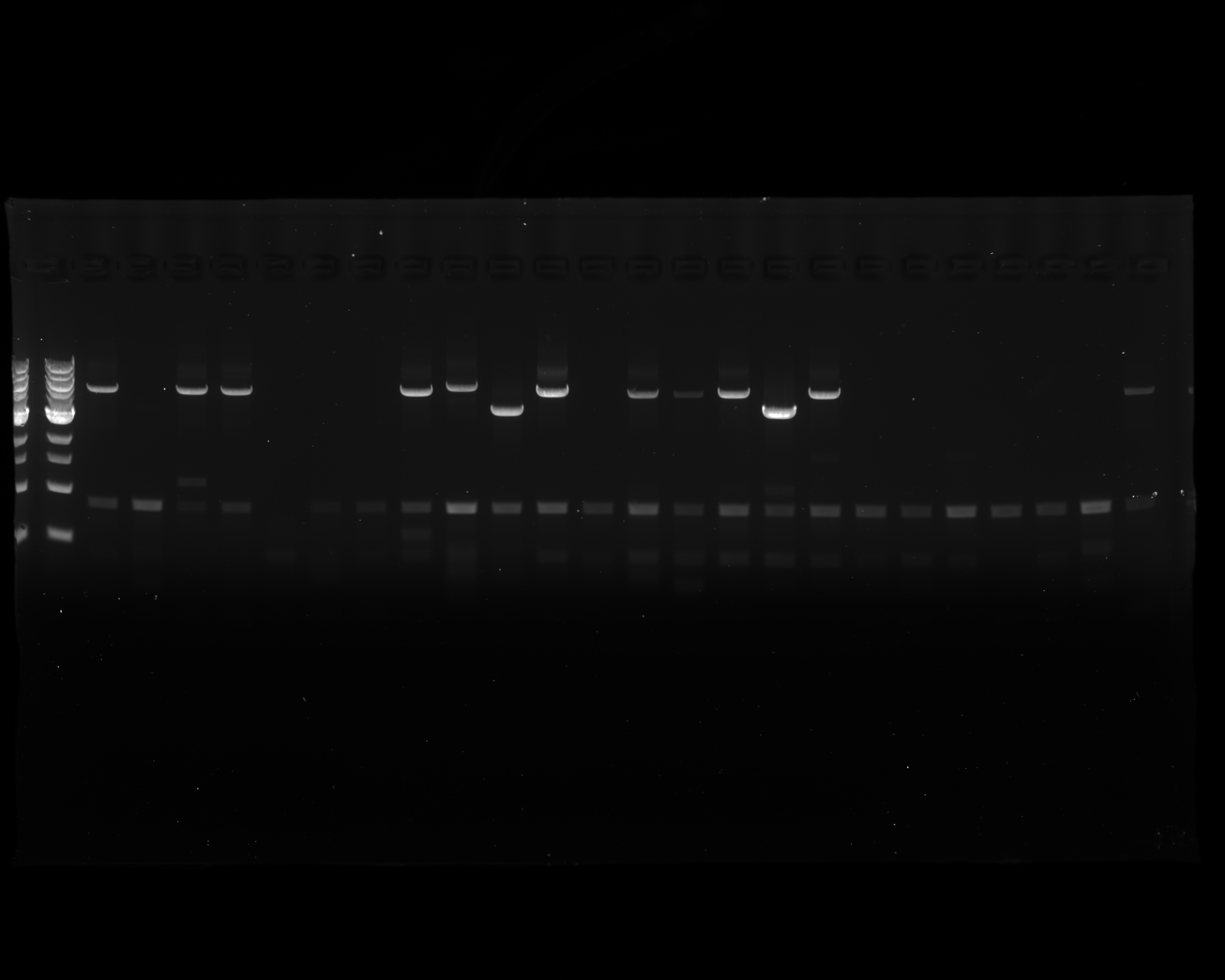

Supplement: Figure 3—figure supplement 3—source data 1. [file elife-95856-fig3-figsupp3-data1.zip › Supplementary Figure 5 - Source Data 1/mESC_Obox4_KO_27_A'+D'.tif]

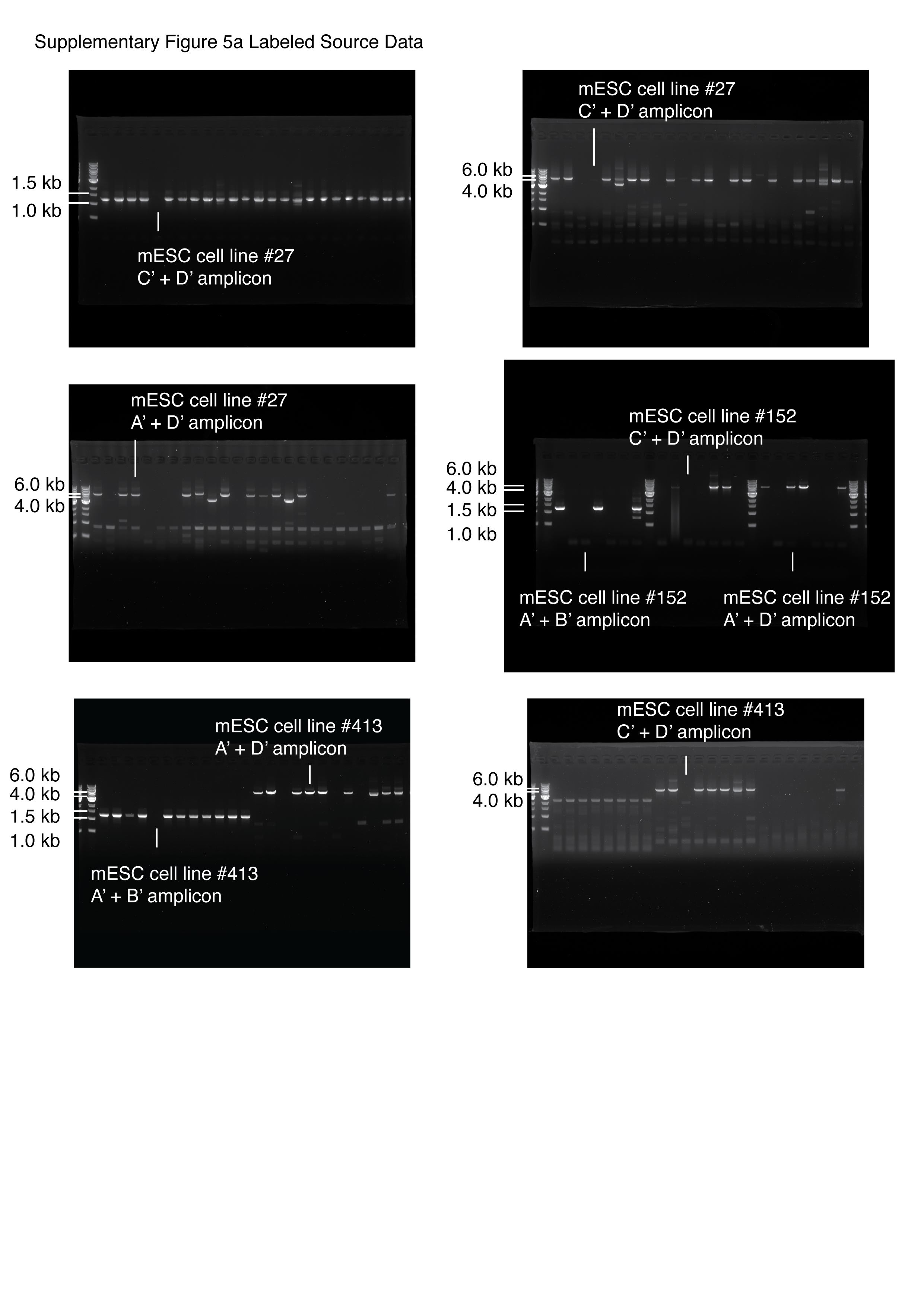

Supplement: Figure 3—figure supplement 3—source data 1. [file elife-95856-fig3-figsupp3-data1.zip › Supplementary Figure 5 - Source Data 1/Supplementary Figure 5a - Labeled Source Data.png]

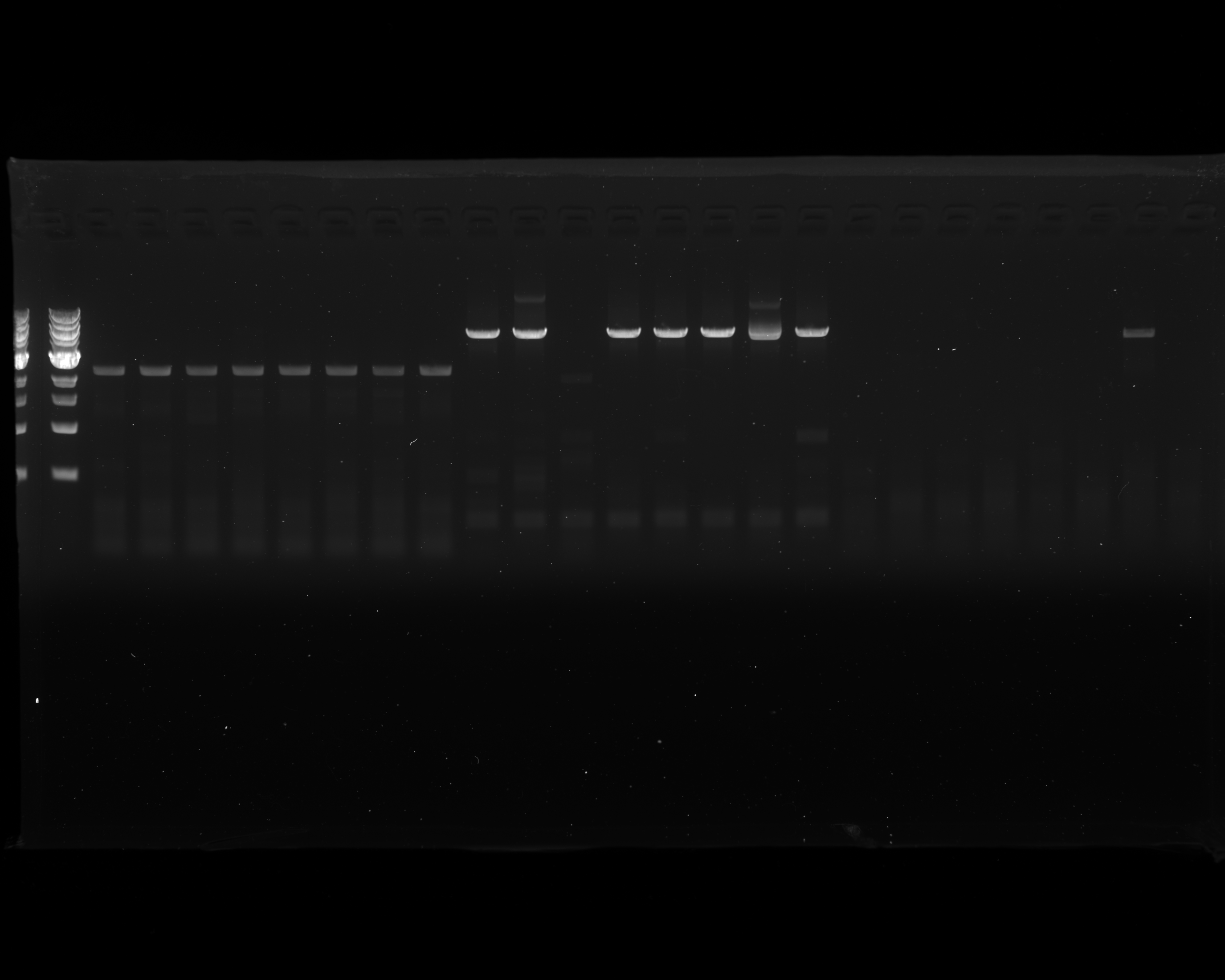

Supplement: Figure 3—figure supplement 3—source data 1. [file elife-95856-fig3-figsupp3-data1.zip › Supplementary Figure 5 - Source Data 1/mESC_Obox4_KO_413_C'+D'.tif]

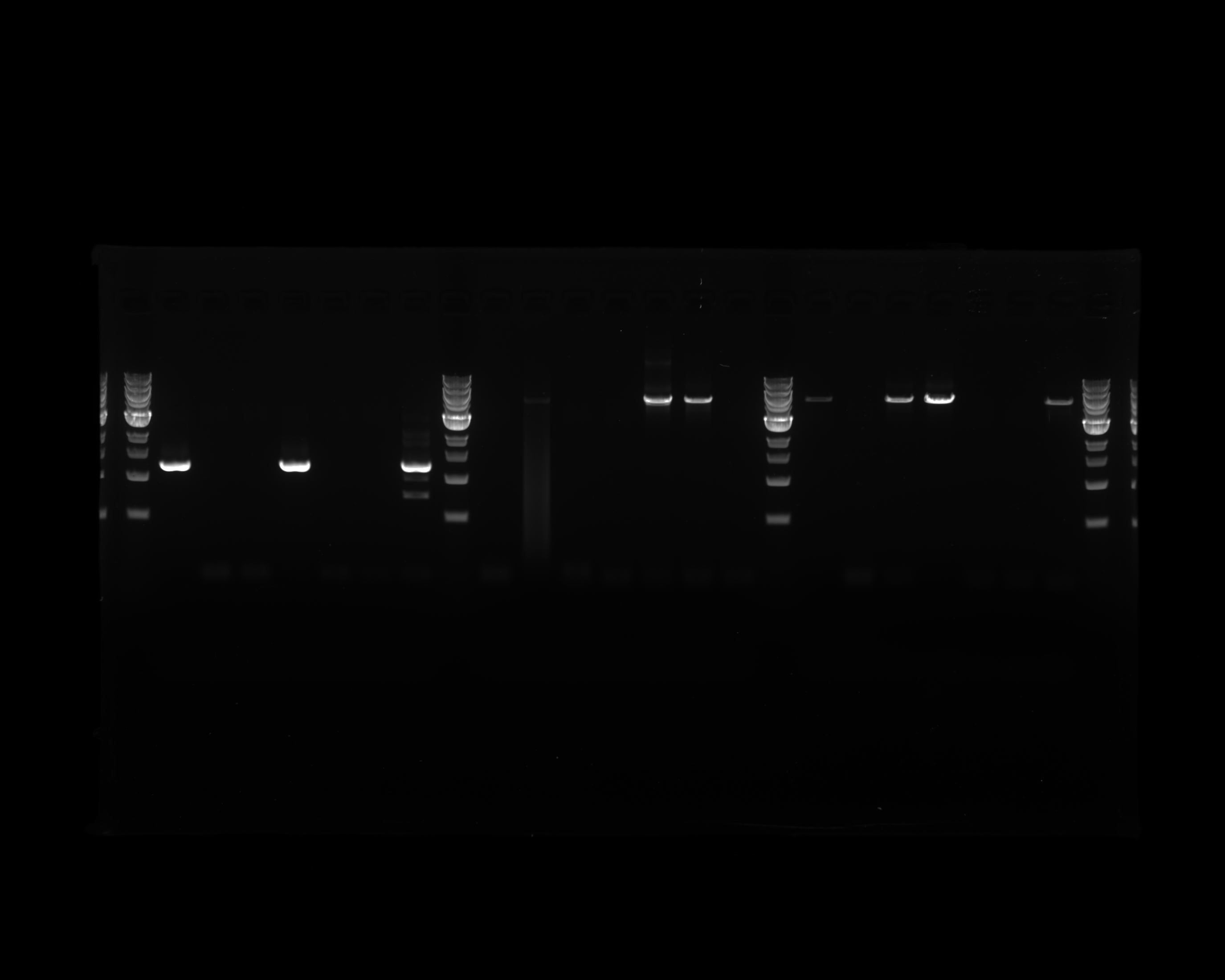

Supplement: Figure 3—figure supplement 3—source data 1. [file elife-95856-fig3-figsupp3-data1.zip › Supplementary Figure 5 - Source Data 1/mESC_Obox4_KO_152.tif]

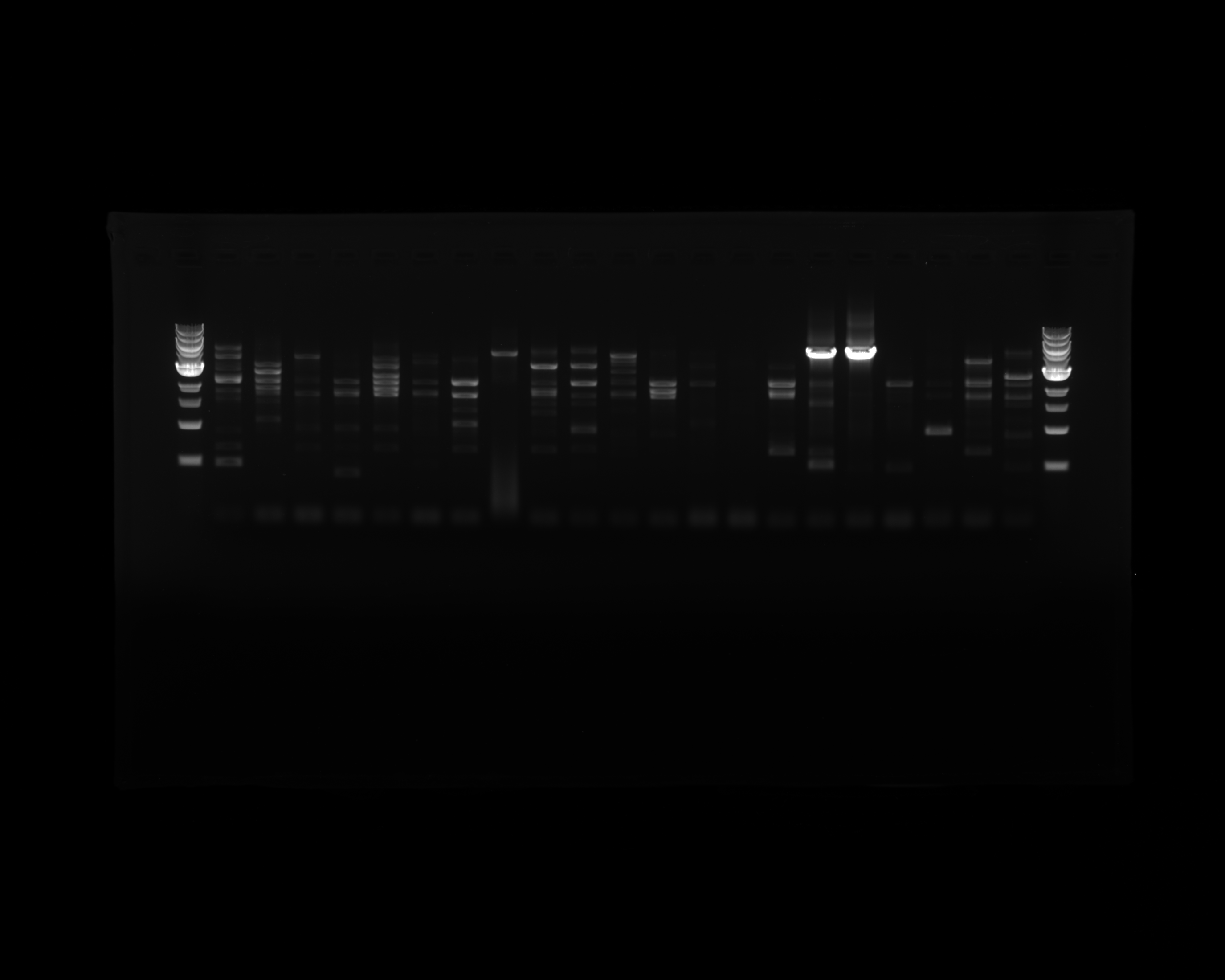

Supplement: Figure 3—figure supplement 3—source data 2. [file elife-95856-fig3-figsupp3-data2.zip › Supplementary Figure 5a - Source Data 2/mESC_Dux_KO_DKO_A+B.tif]

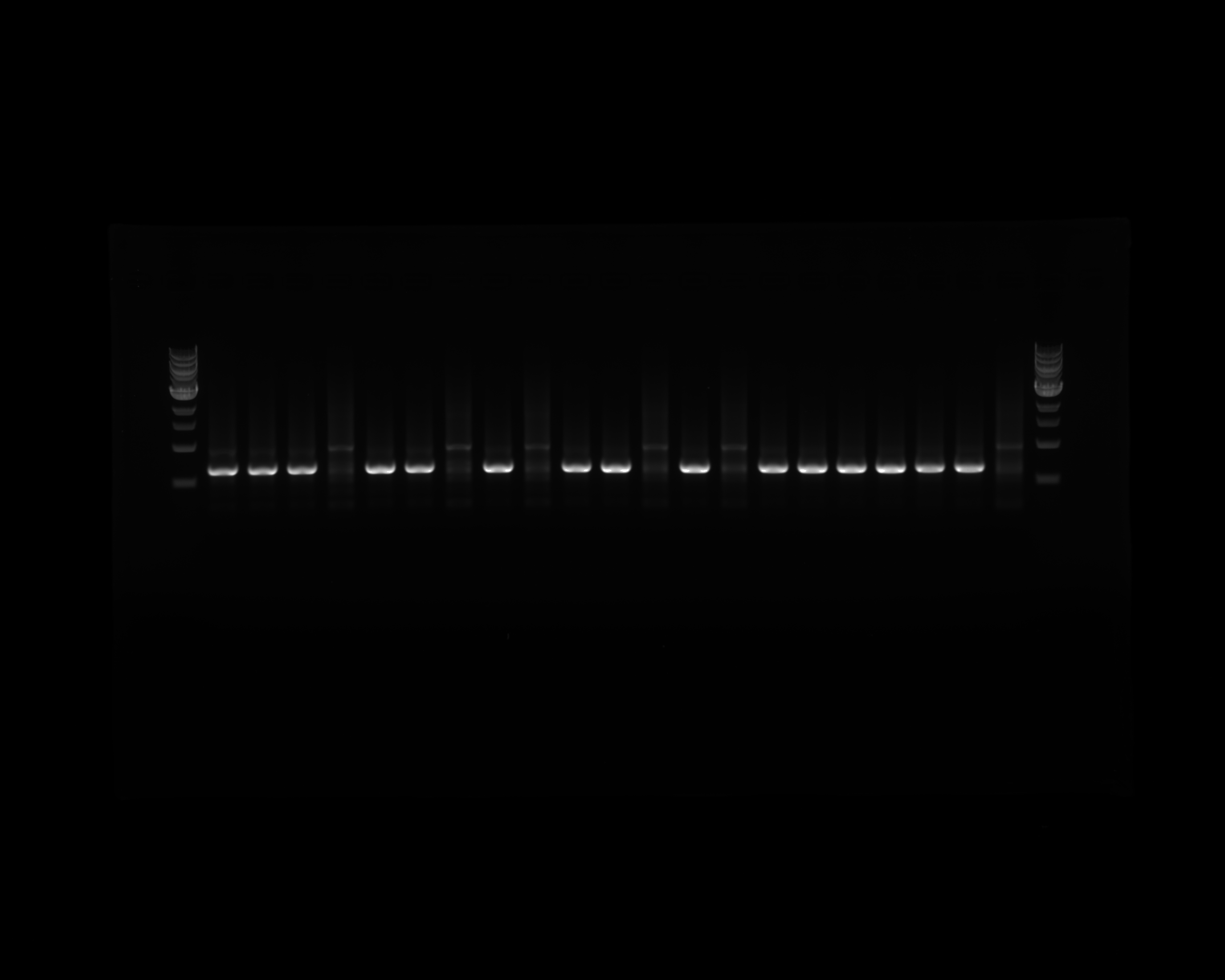

Supplement: Figure 3—figure supplement 3—source data 2. [file elife-95856-fig3-figsupp3-data2.zip › Supplementary Figure 5a - Source Data 2/mESC_Dux_KO_DKO_E+F.tif]

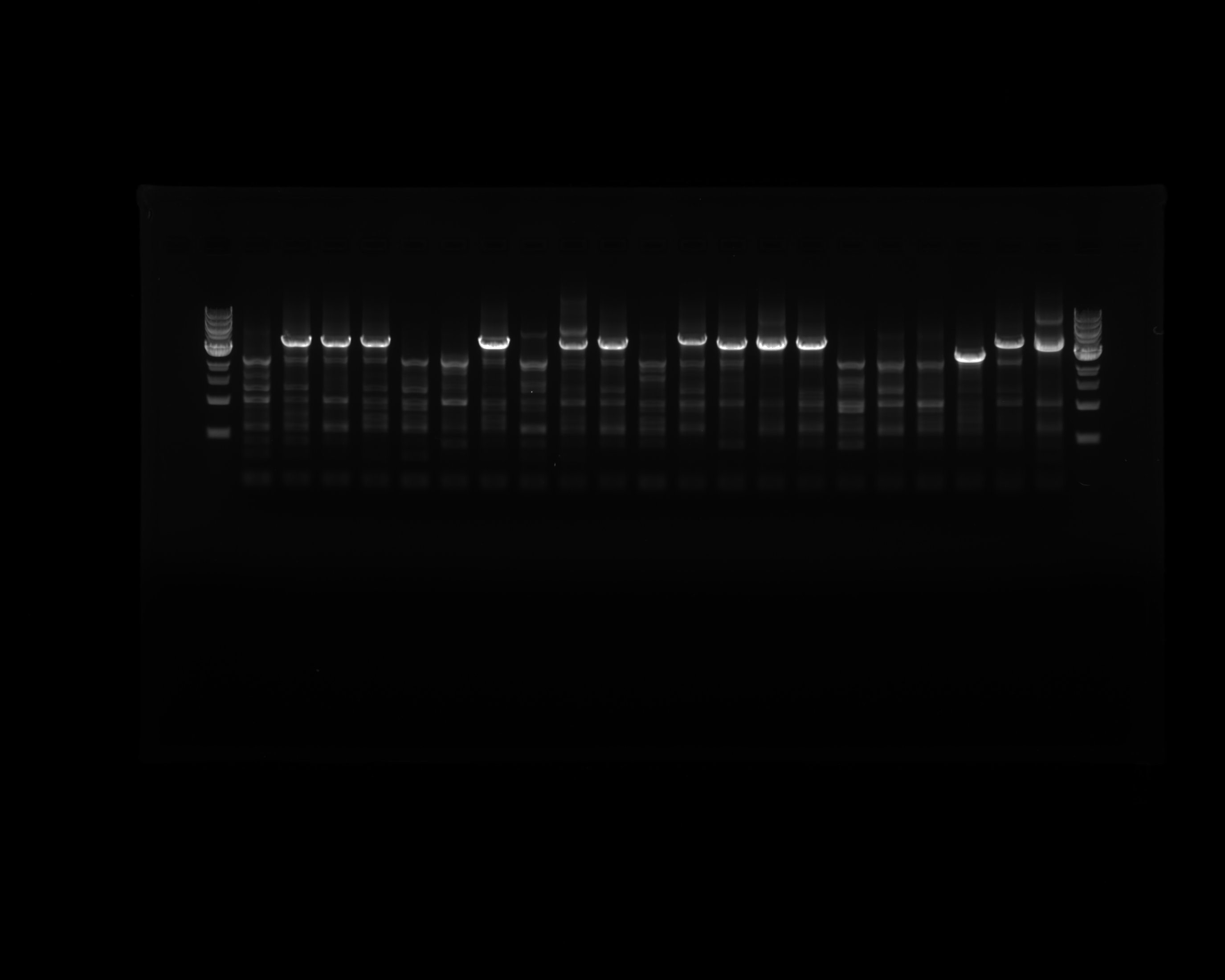

Supplement: Figure 3—figure supplement 3—source data 2. [file elife-95856-fig3-figsupp3-data2.zip › Supplementary Figure 5a - Source Data 2/mESC_Dux_KO_DKO_A+D.tif]

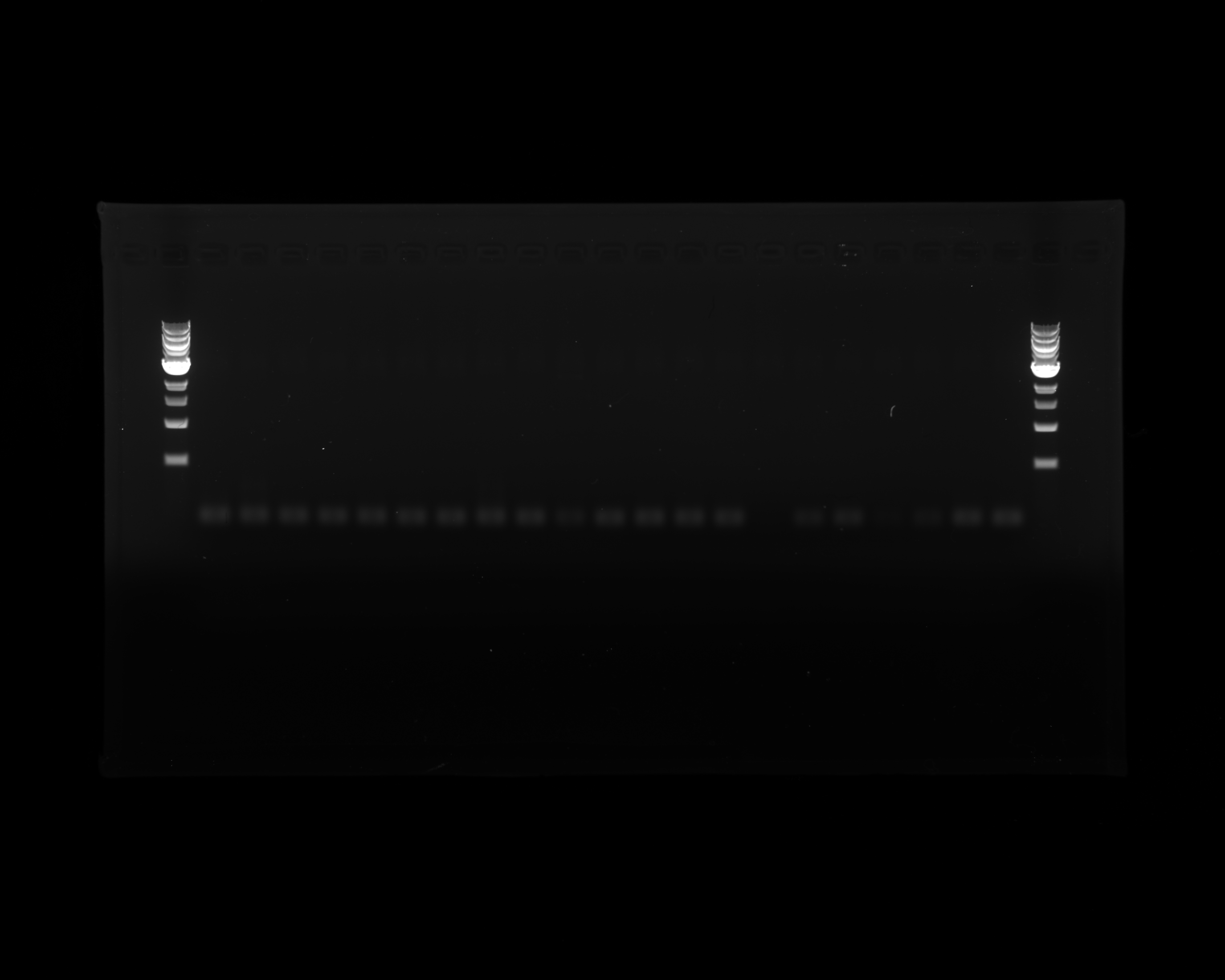

Supplement: Figure 3—figure supplement 3—source data 2. [file elife-95856-fig3-figsupp3-data2.zip › Supplementary Figure 5a - Source Data 2/mESC_Dux_KO_DKO_C+D.tif]

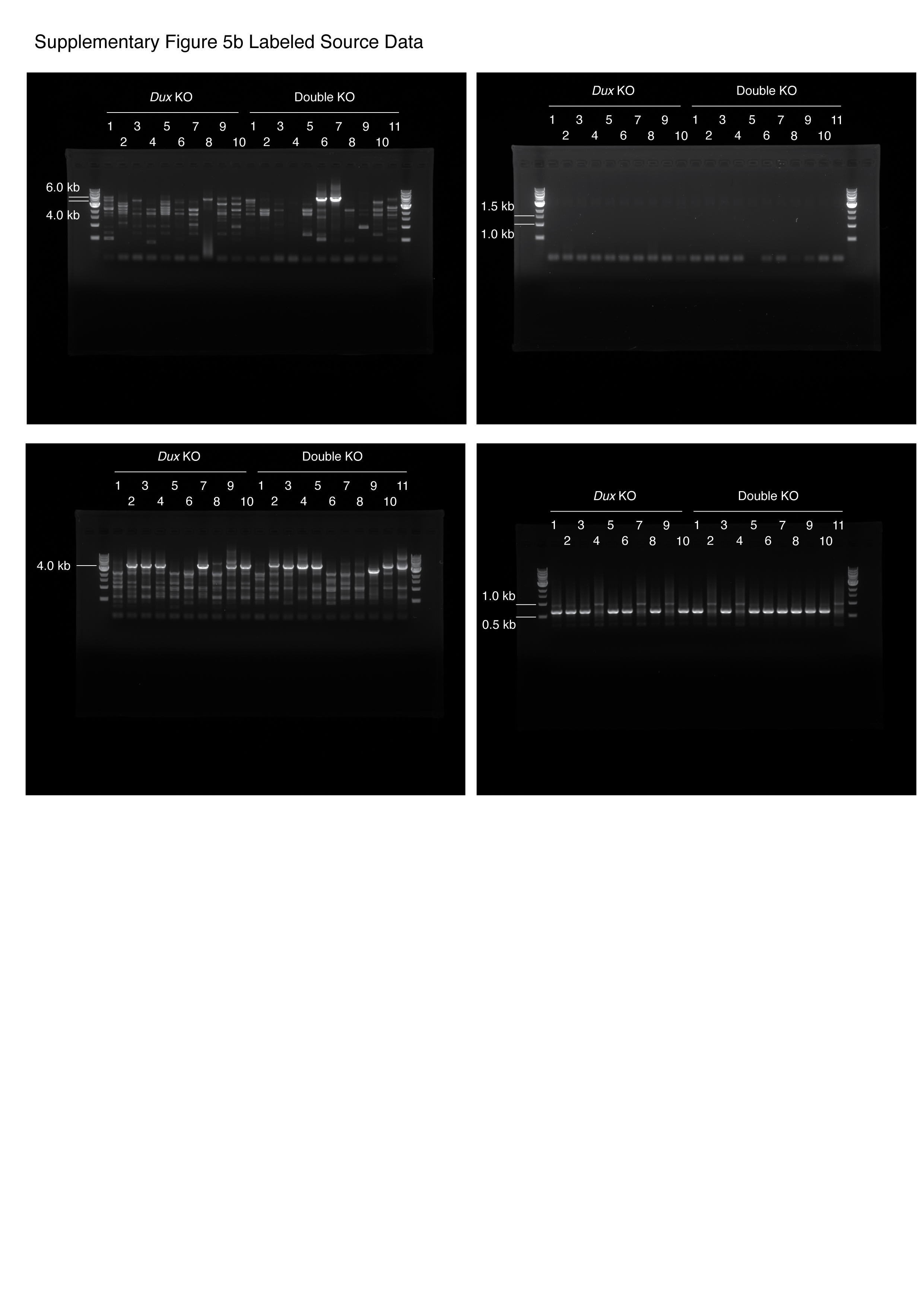

Supplement: Figure 3—figure supplement 3—source data 2. [file elife-95856-fig3-figsupp3-data2.zip › Supplementary Figure 5a - Source Data 2/Supplementary Figure 5b - Labeled Source Data.png]

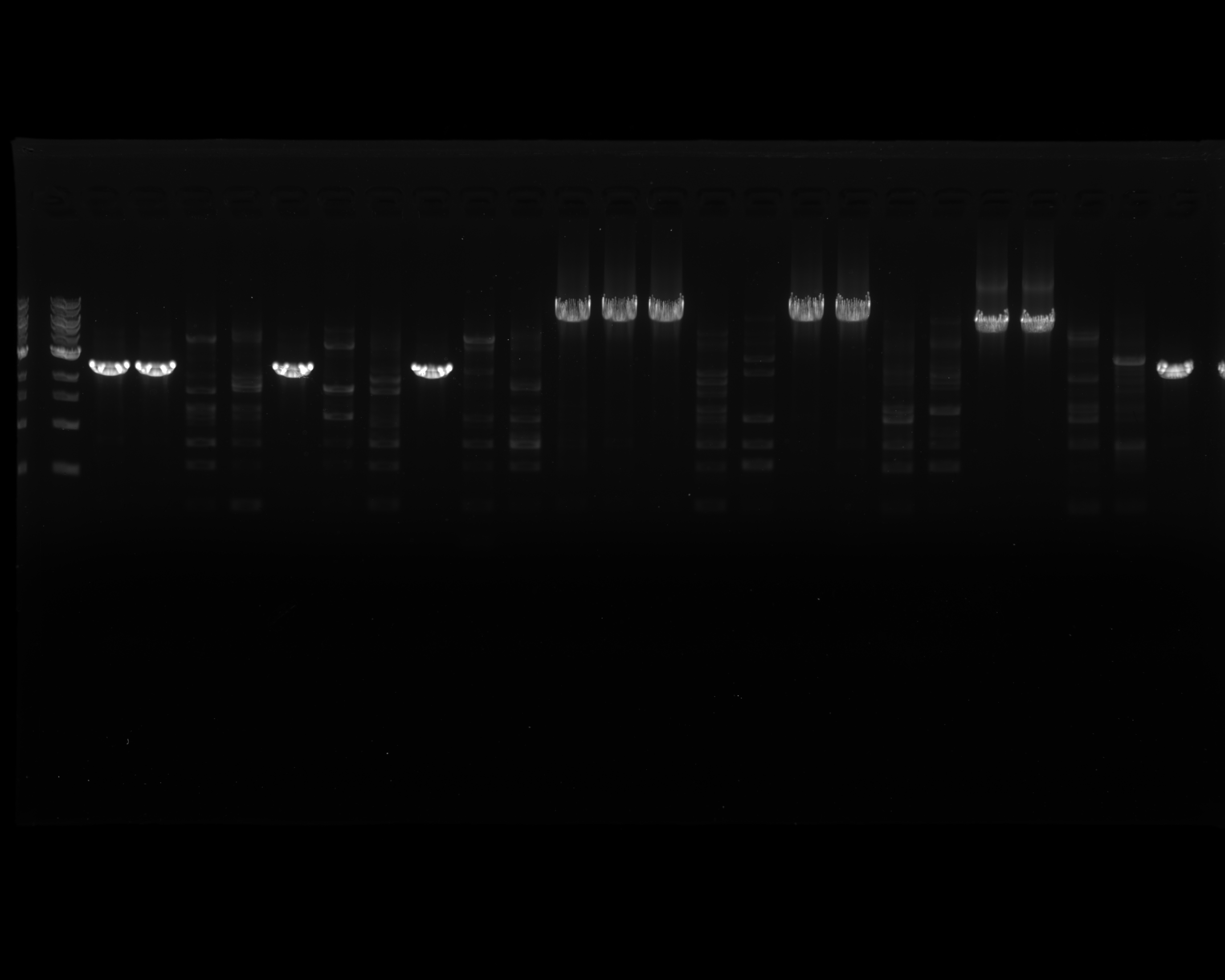

Supplement: Figure 4—figure supplement 1—source data 1. [file elife-95856-fig4-figsupp1-data1.zip › Supplementary Figure 6 - Source Data 1/Mice_F1_A'+D'_131to152.tif]

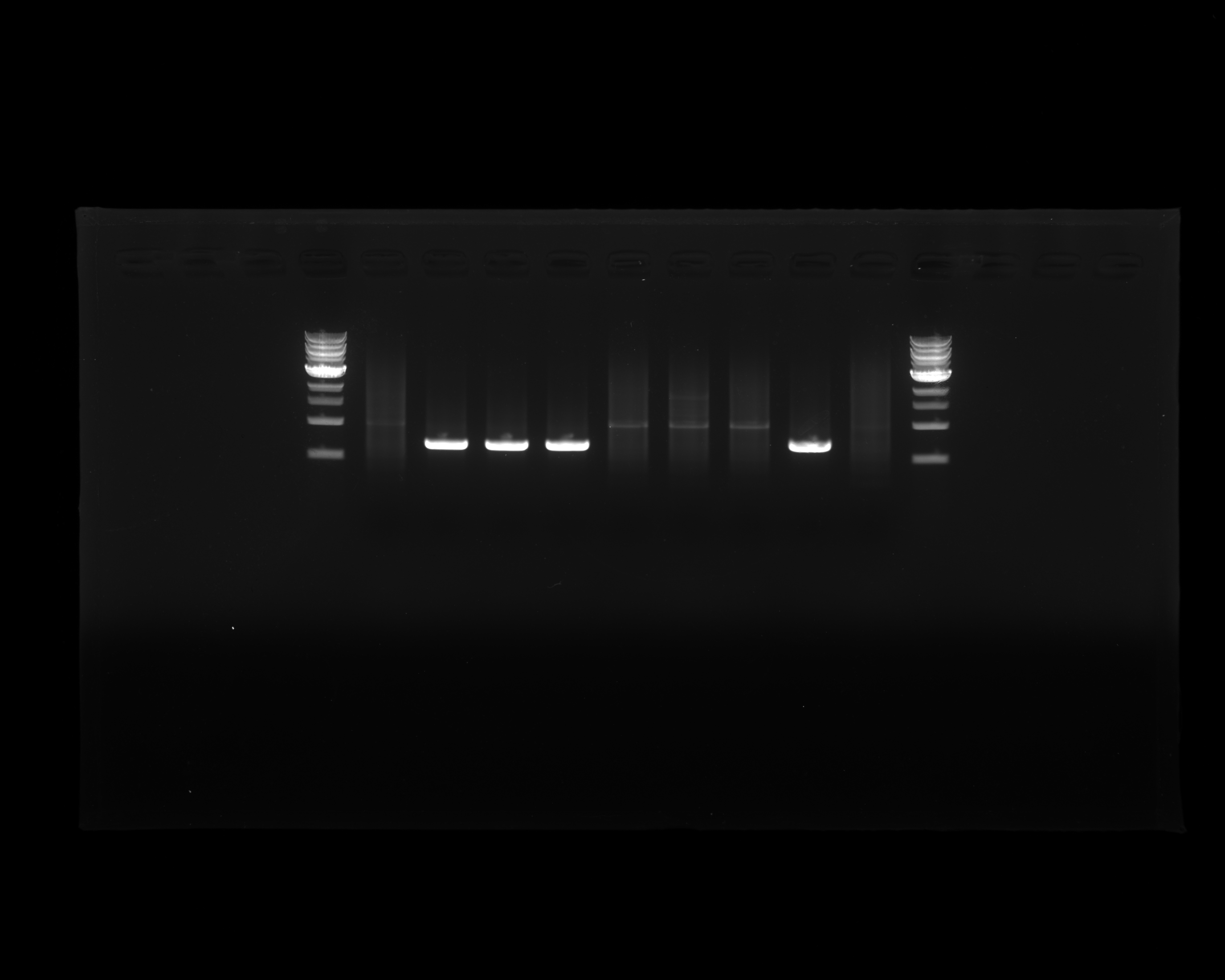

Supplement: Figure 4—figure supplement 1—source data 1. [file elife-95856-fig4-figsupp1-data1.zip › Supplementary Figure 6 - Source Data 1/Mice_F1_E+F_132to146.tif]

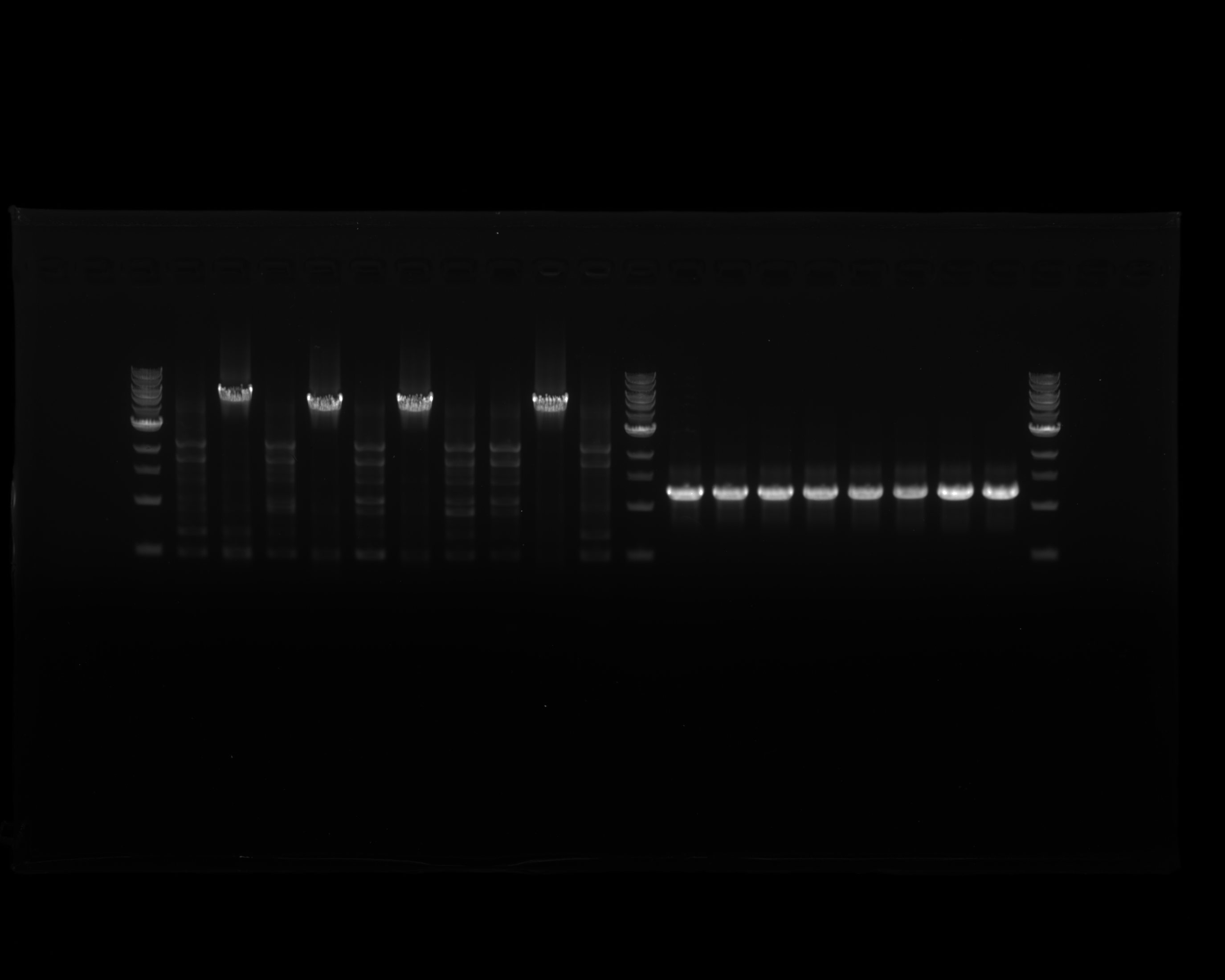

Supplement: Figure 4—figure supplement 1—source data 1. [file elife-95856-fig4-figsupp1-data1.zip › Supplementary Figure 6 - Source Data 1/Mice_F1_A'+B'_A+B_108to129.tif]

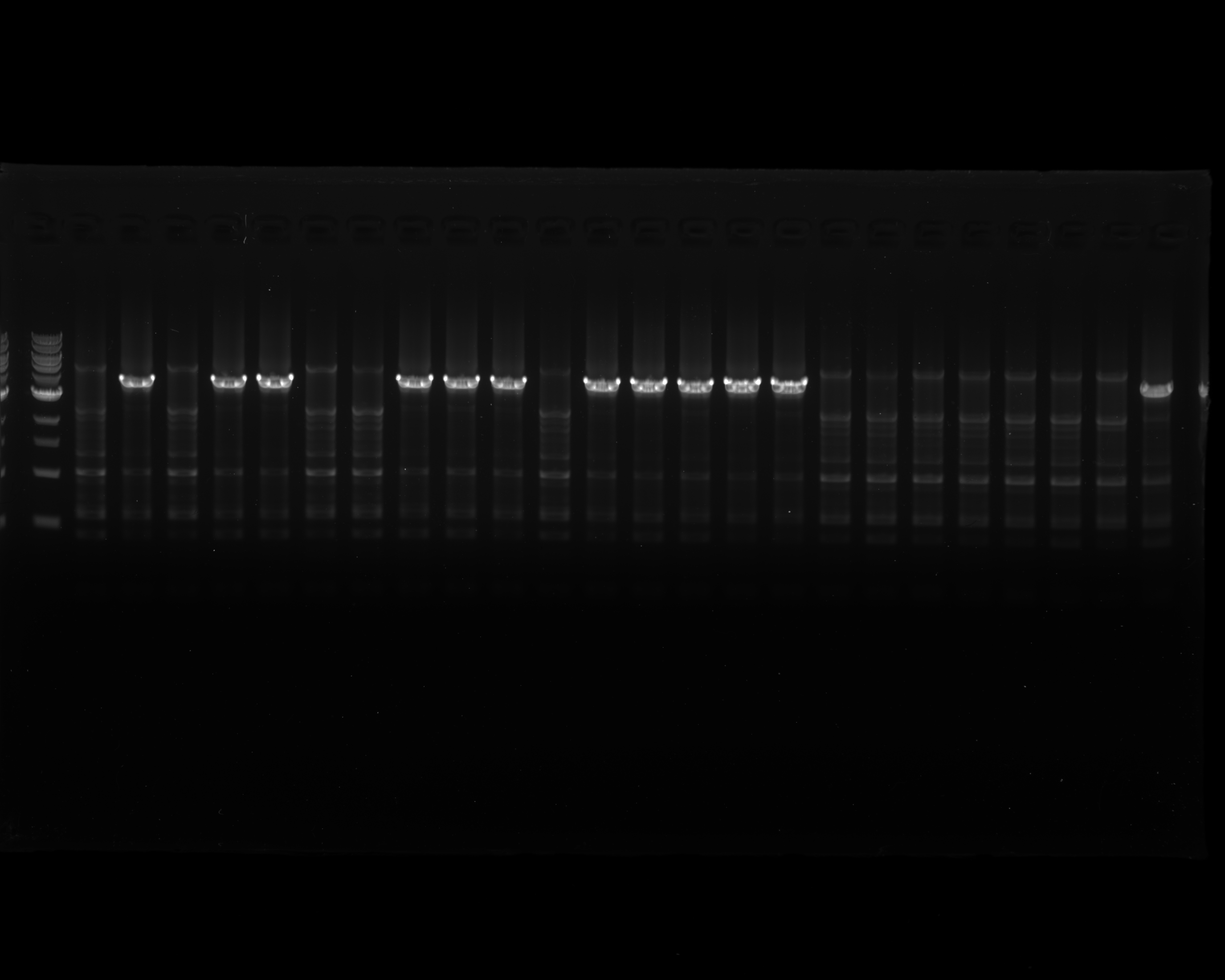

Supplement: Figure 4—figure supplement 1—source data 1. [file elife-95856-fig4-figsupp1-data1.zip › Supplementary Figure 6 - Source Data 1/Mice_F1_A+D_131to152.tif]

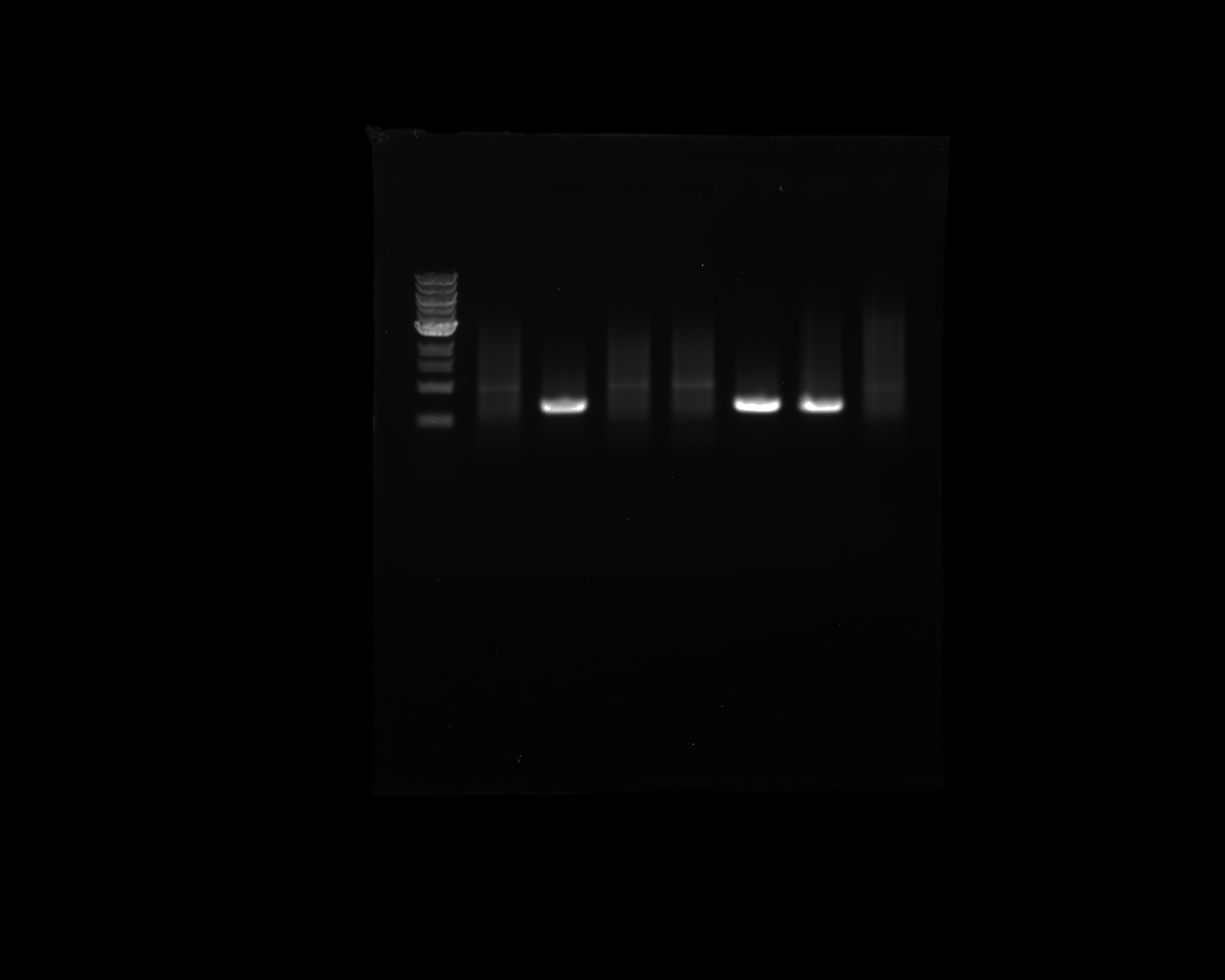

Supplement: Figure 4—figure supplement 1—source data 1. [file elife-95856-fig4-figsupp1-data1.zip › Supplementary Figure 6 - Source Data 1/Mice_F1_E+F_109to128.tif]

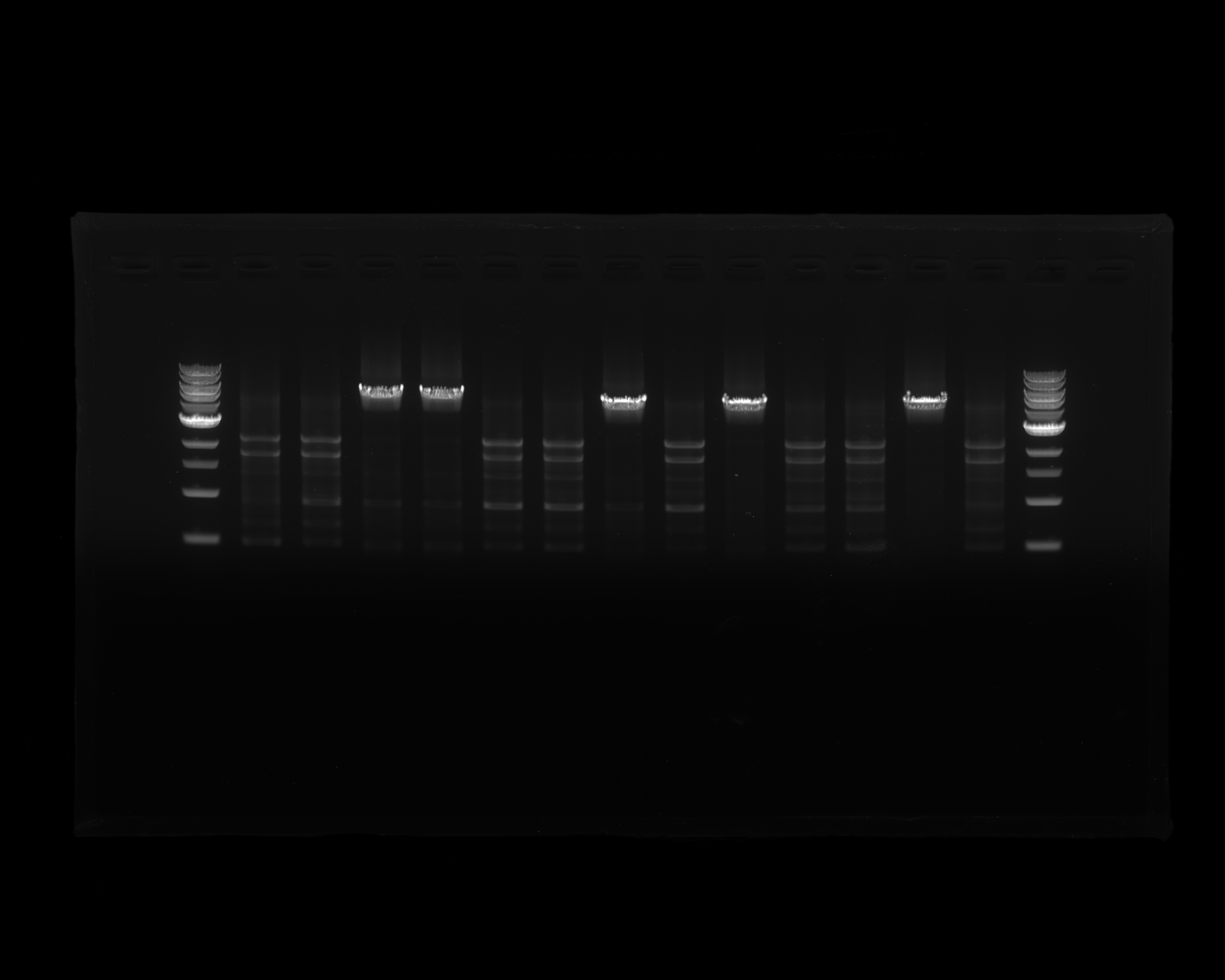

Supplement: Figure 4—figure supplement 1—source data 1. [file elife-95856-fig4-figsupp1-data1.zip › Supplementary Figure 6 - Source Data 1/Mice_F1_A+B_132to146.tif]

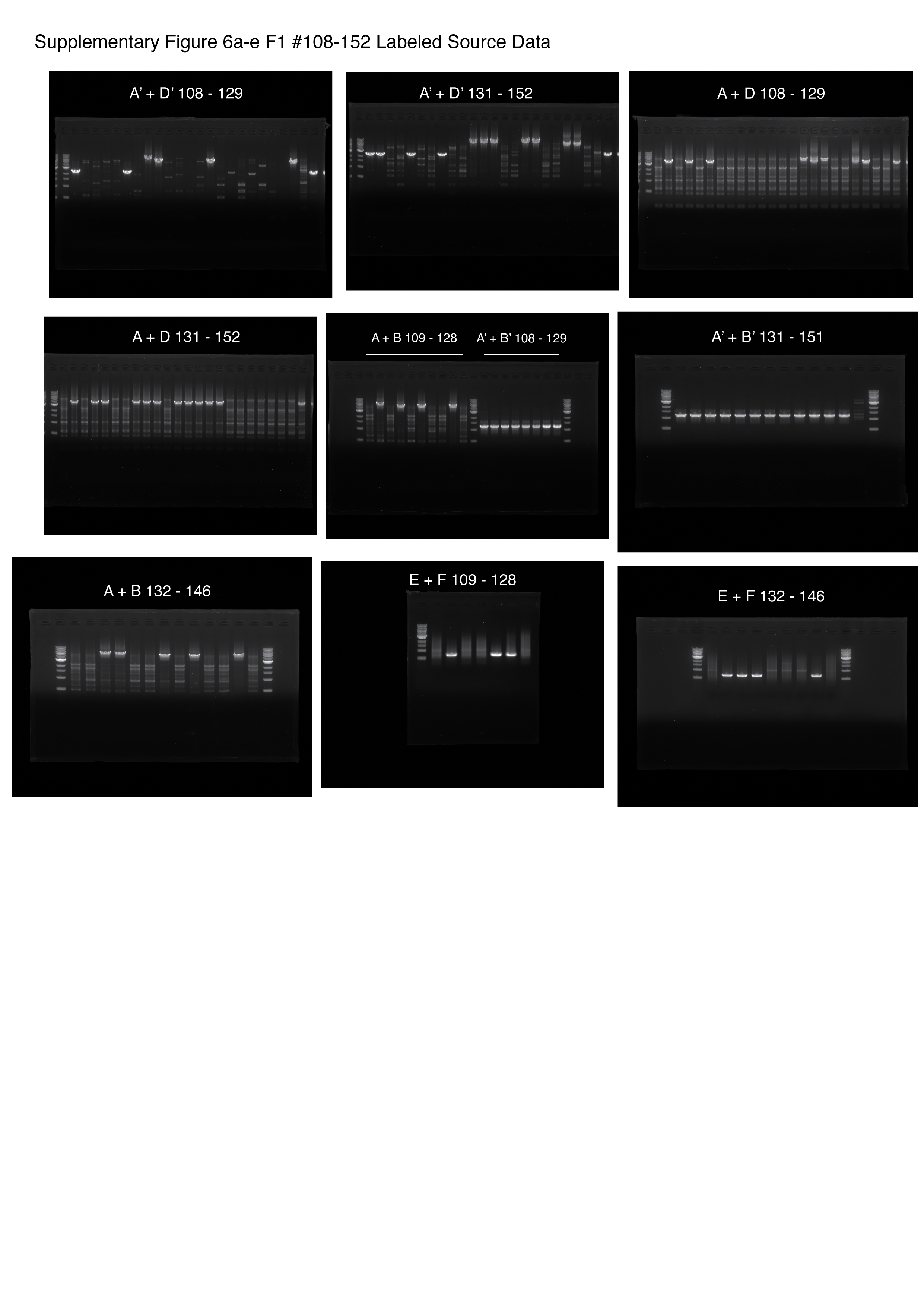

Supplement: Figure 4—figure supplement 1—source data 1. [file elife-95856-fig4-figsupp1-data1.zip › Supplementary Figure 6 - Source Data 1/Supplementary Figure 6a-e F1 #108-152 - Labeled Source Data.png]

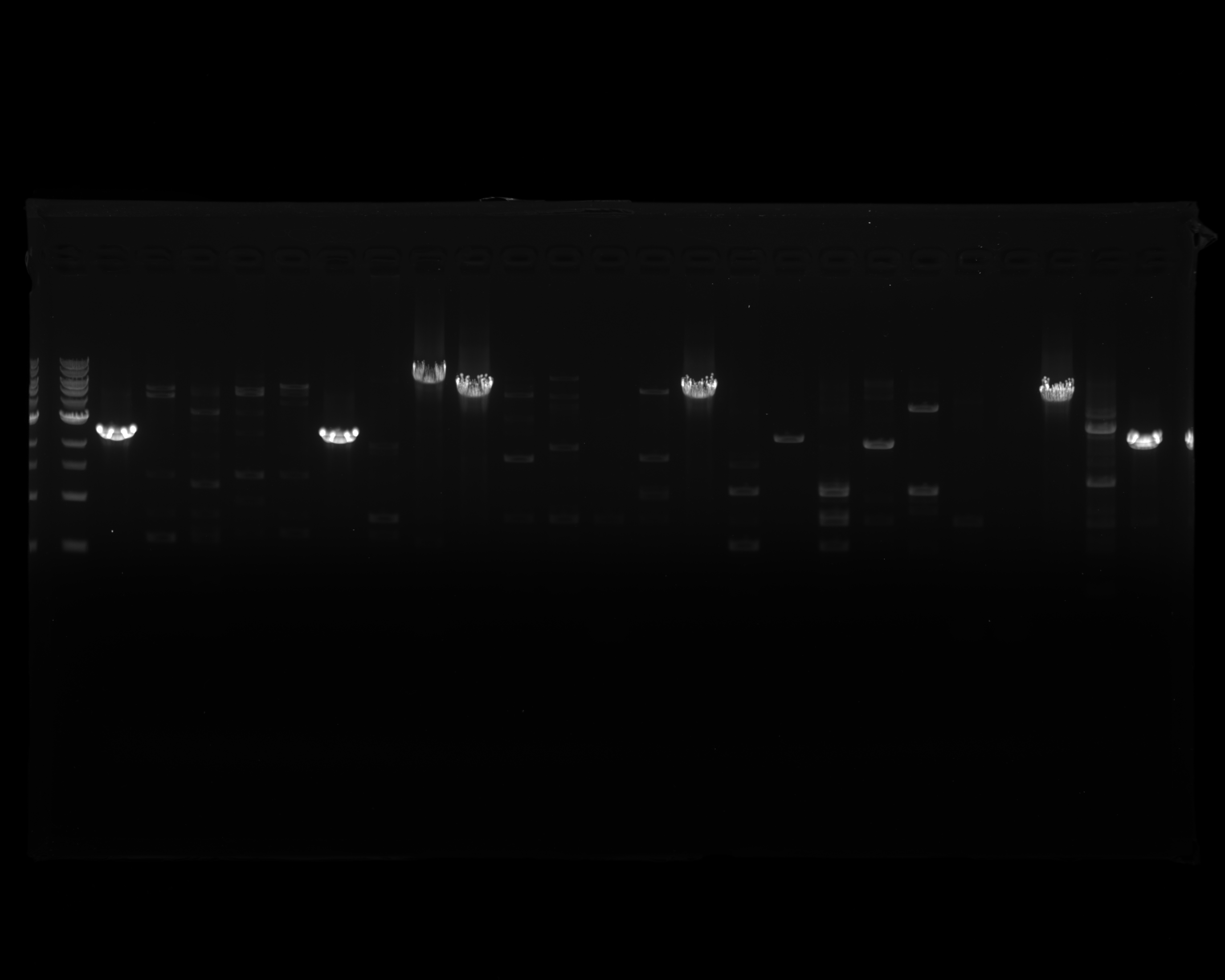

Supplement: Figure 4—figure supplement 1—source data 1. [file elife-95856-fig4-figsupp1-data1.zip › Supplementary Figure 6 - Source Data 1/Mice_F1_A'+D'_108to129.tif]

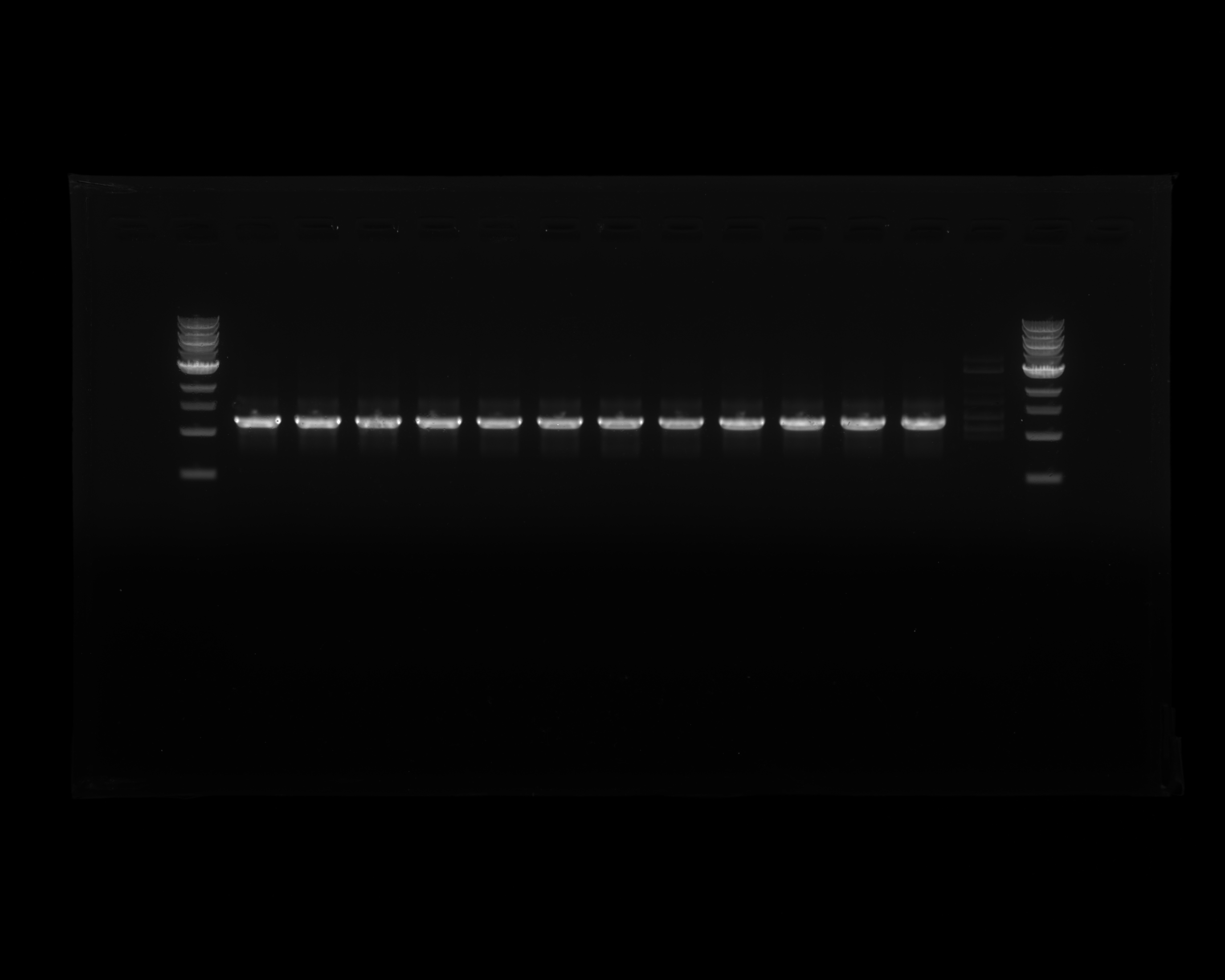

Supplement: Figure 4—figure supplement 1—source data 1. [file elife-95856-fig4-figsupp1-data1.zip › Supplementary Figure 6 - Source Data 1/Mice_F1_A'+B'_131to151.tif]

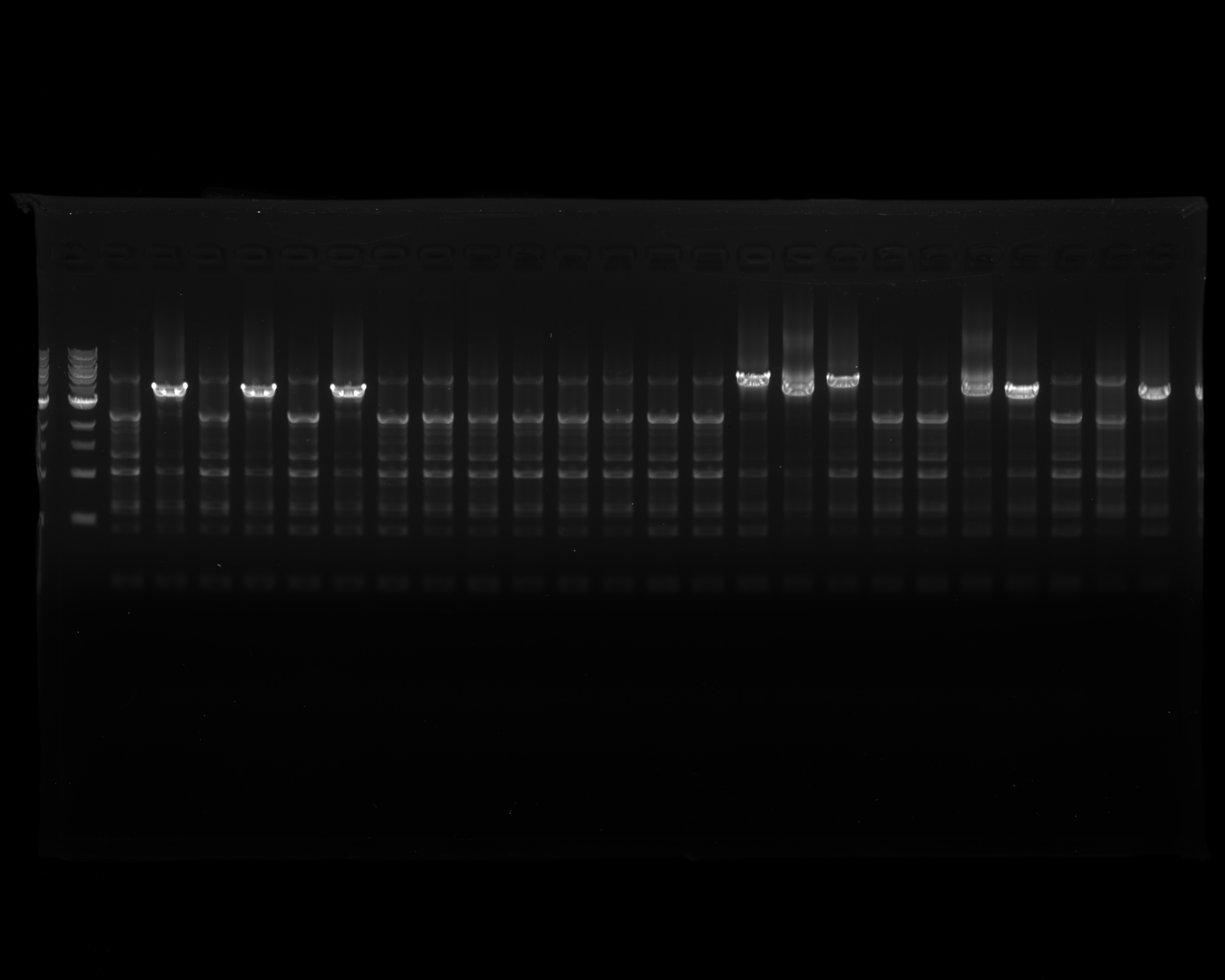

Supplement: Figure 4—figure supplement 1—source data 1. [file elife-95856-fig4-figsupp1-data1.zip › Supplementary Figure 6 - Source Data 1/Mice_F1_A+D_108to129.tif]

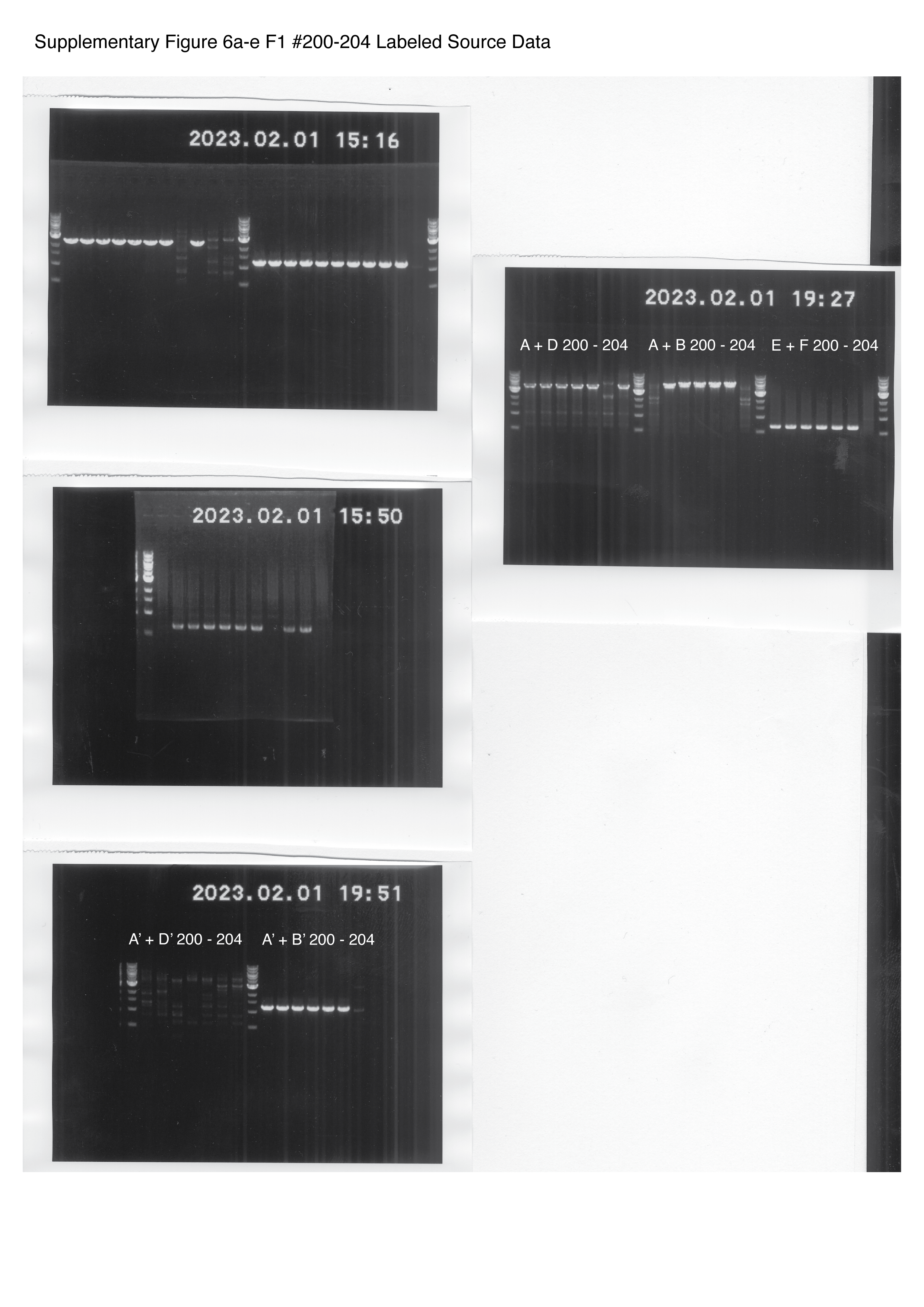

Supplement: Figure 4—figure supplement 1—source data 2. [file elife-95856-fig4-figsupp1-data2.zip › Supplementary Figure 6 - Source Data 2/Supplementary Figure 6a-e F1 #200-204 - Labeled Source Data.png]

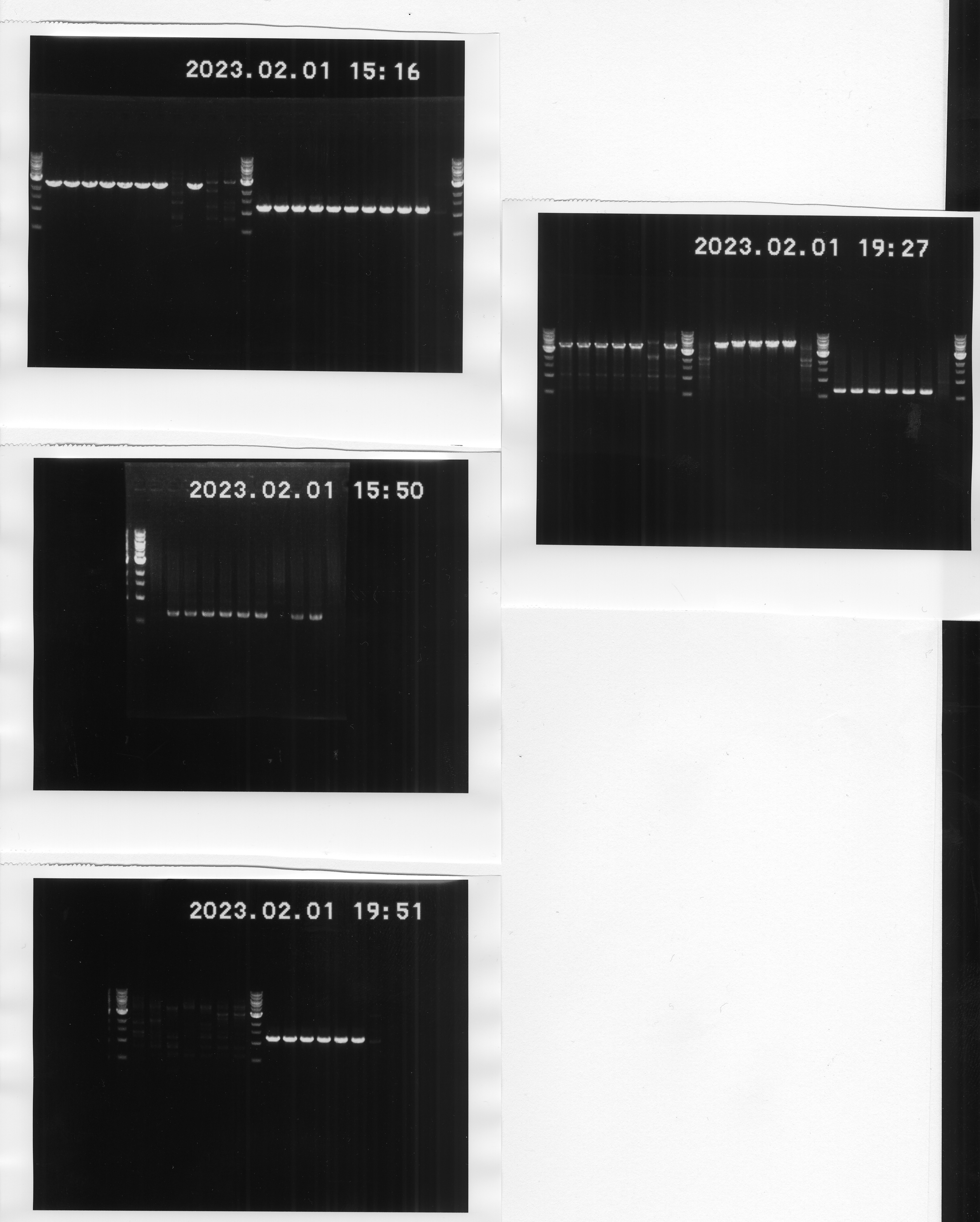

Supplement: Figure 4—figure supplement 1—source data 2. [file elife-95856-fig4-figsupp1-data2.zip › Supplementary Figure 6 - Source Data 2/Mice_F1_200to204.tif]

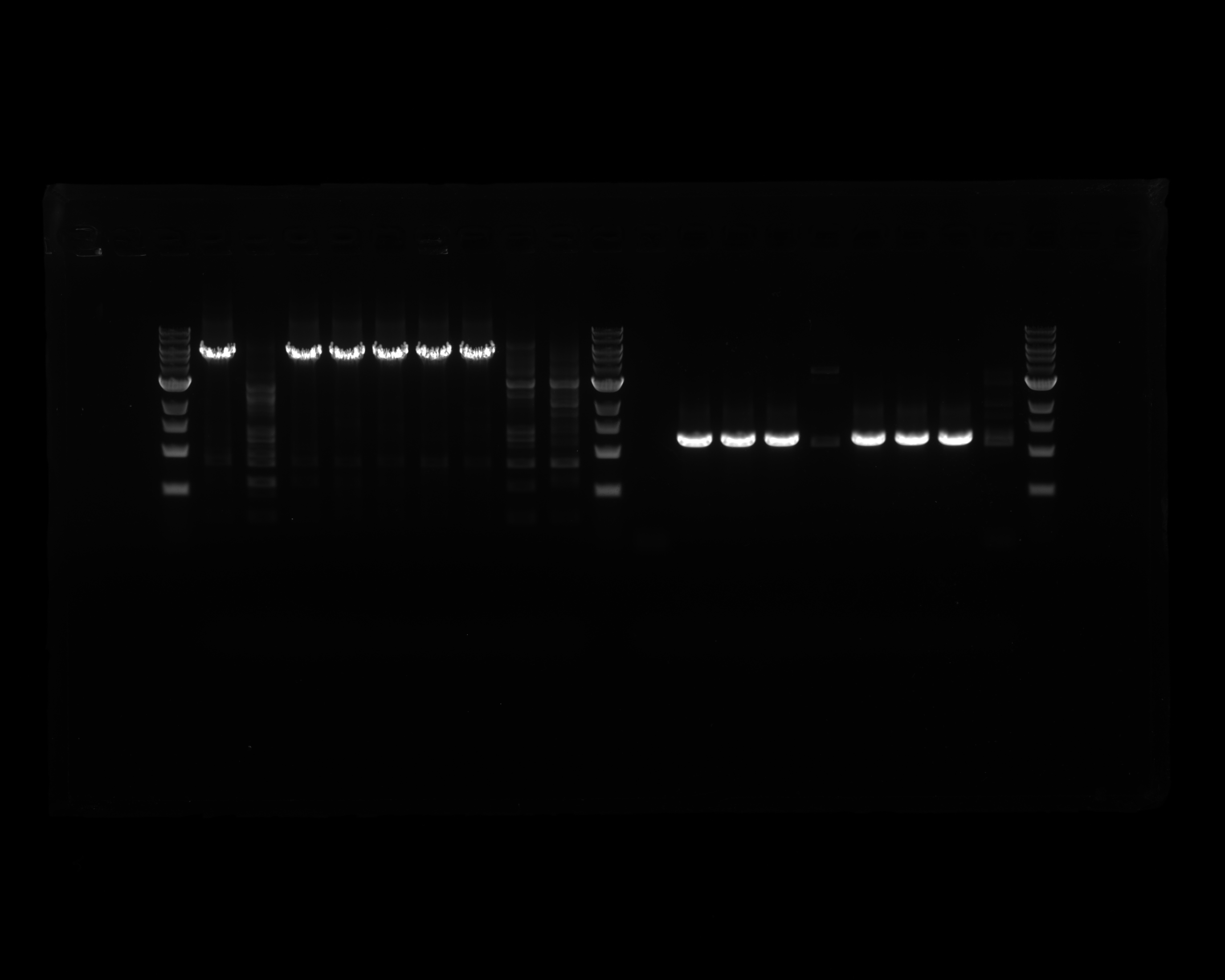

Supplement: Figure 4—figure supplement 2—source data 1. [file elife-95856-fig4-figsupp2-data1.zip › Supplementary Figure 7 - Source Data/Mice_F2_180to186.tif]

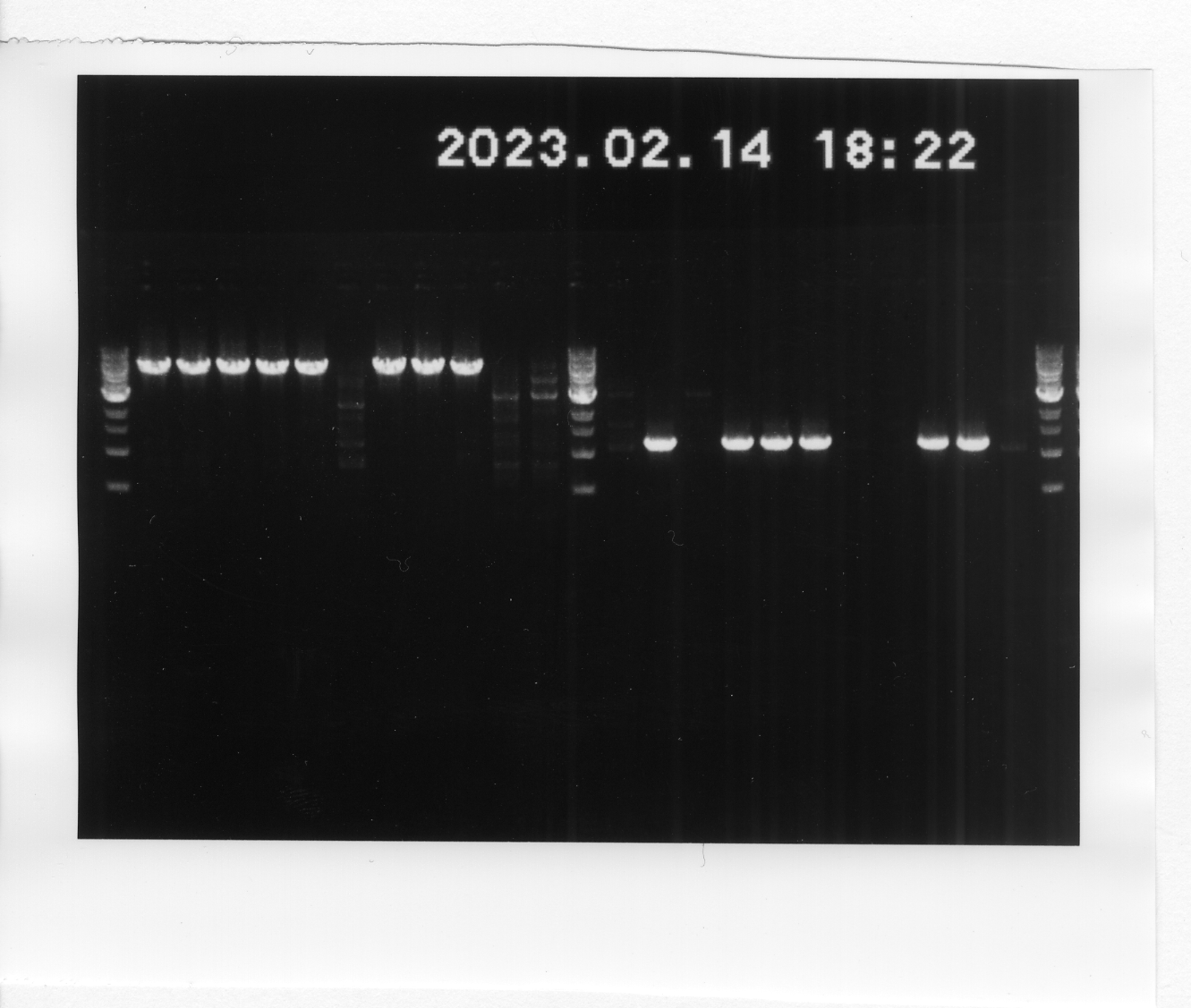

Supplement: Figure 4—figure supplement 2—source data 1. [file elife-95856-fig4-figsupp2-data1.zip › Supplementary Figure 7 - Source Data/Mice_F2_205to213.tif]

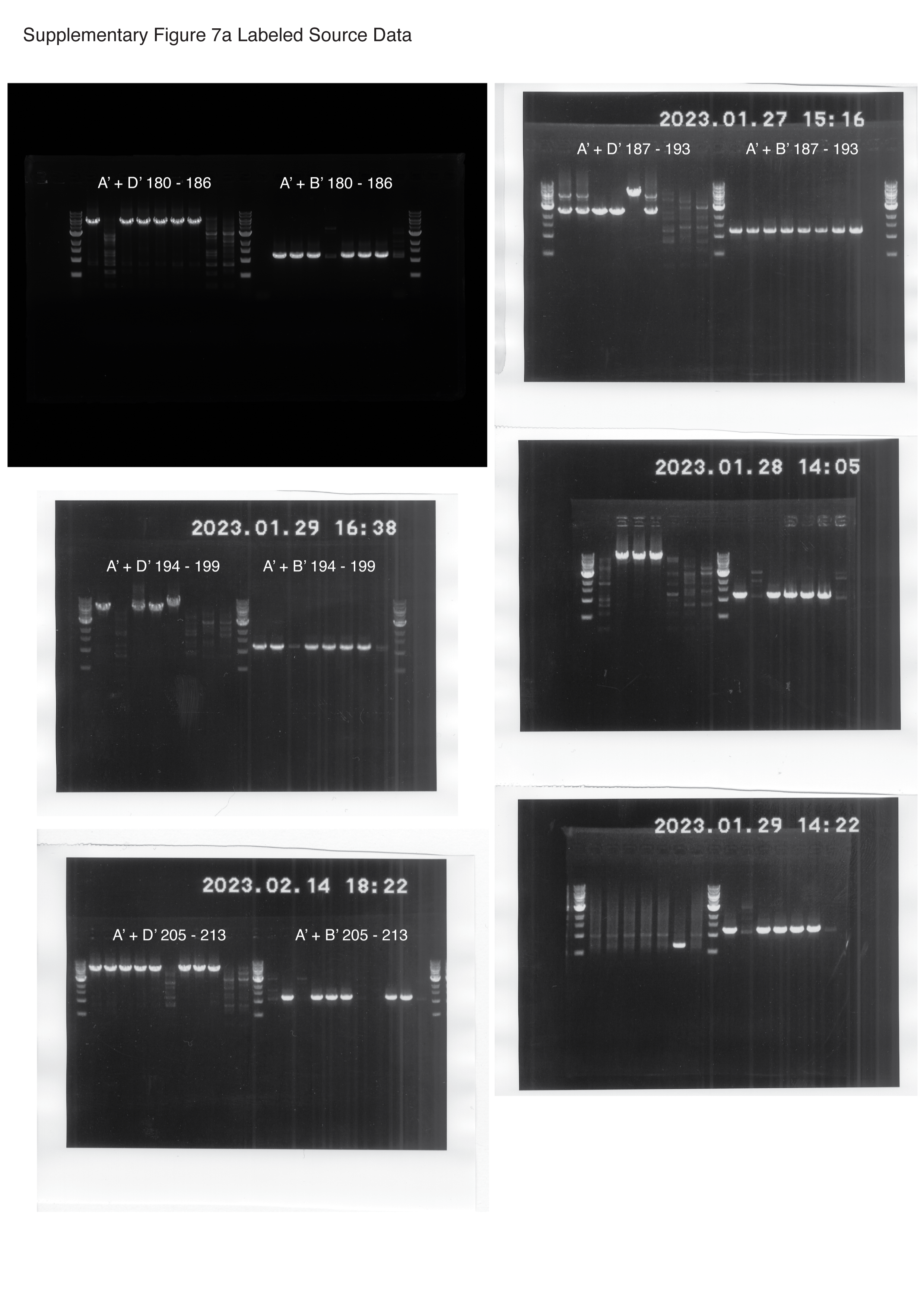

Supplement: Figure 4—figure supplement 2—source data 1. [file elife-95856-fig4-figsupp2-data1.zip › Supplementary Figure 7 - Source Data/Supplementary Figure 7a - Labeled Source Data.png]

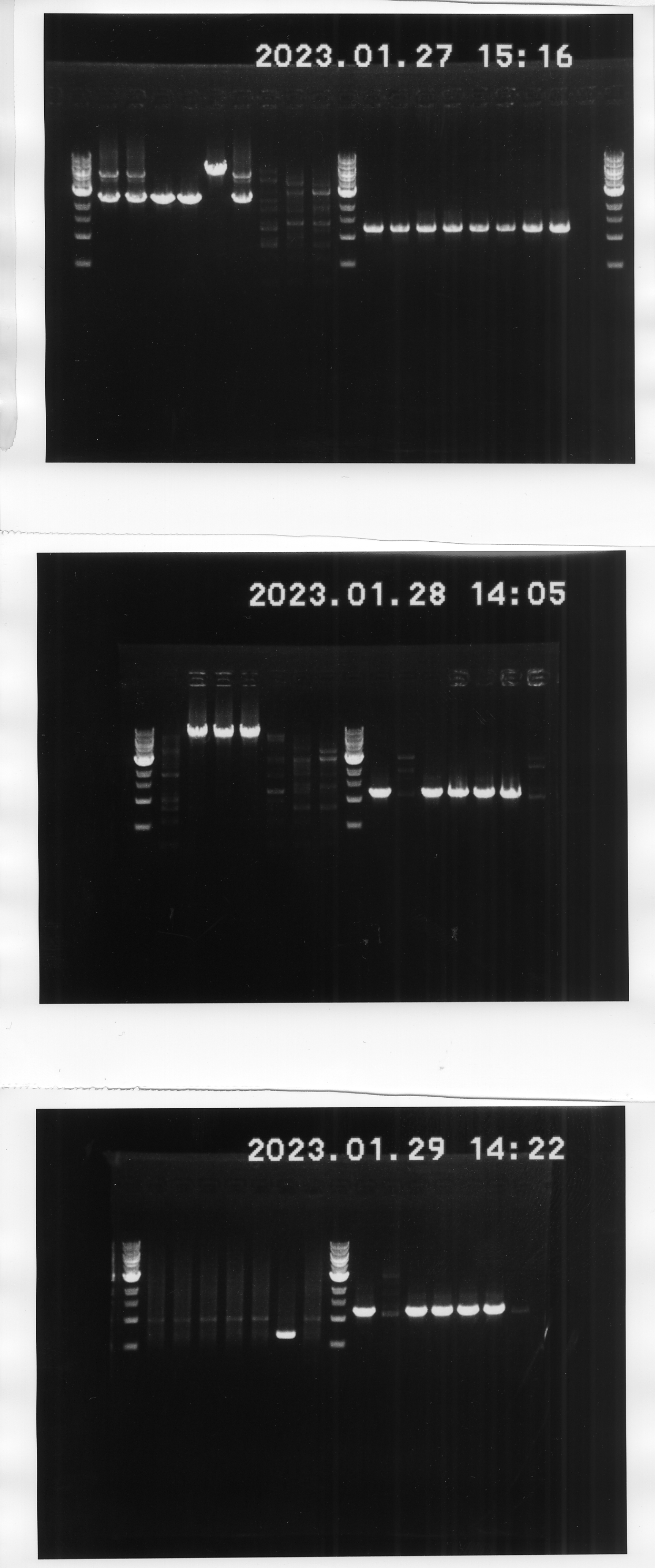

Supplement: Figure 4—figure supplement 2—source data 1. [file elife-95856-fig4-figsupp2-data1.zip › Supplementary Figure 7 - Source Data/Mice_F2_187to193.tif]

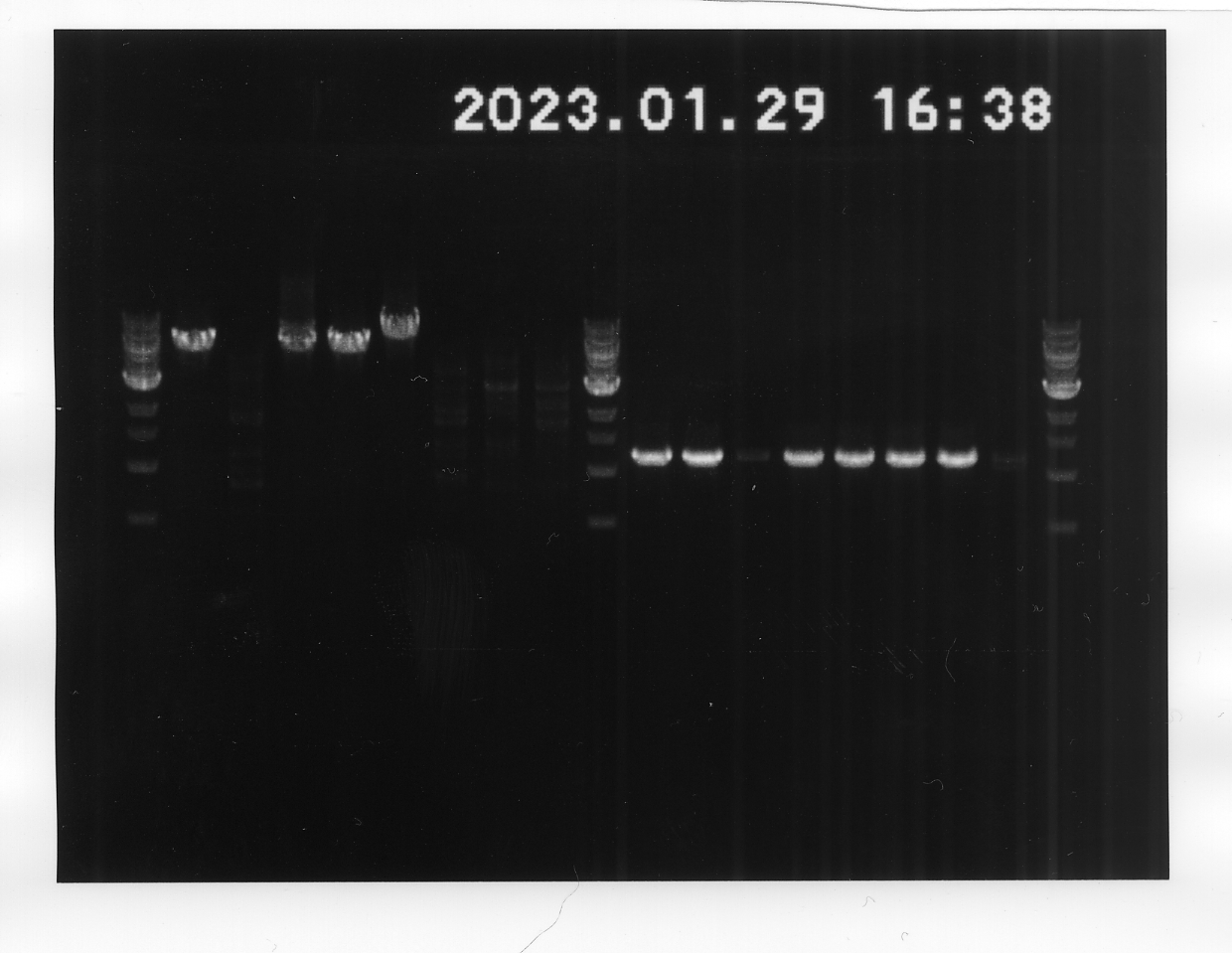

Supplement: Figure 4—figure supplement 2—source data 1. [file elife-95856-fig4-figsupp2-data1.zip › Supplementary Figure 7 - Source Data/Mice_F2_194to199.tif]

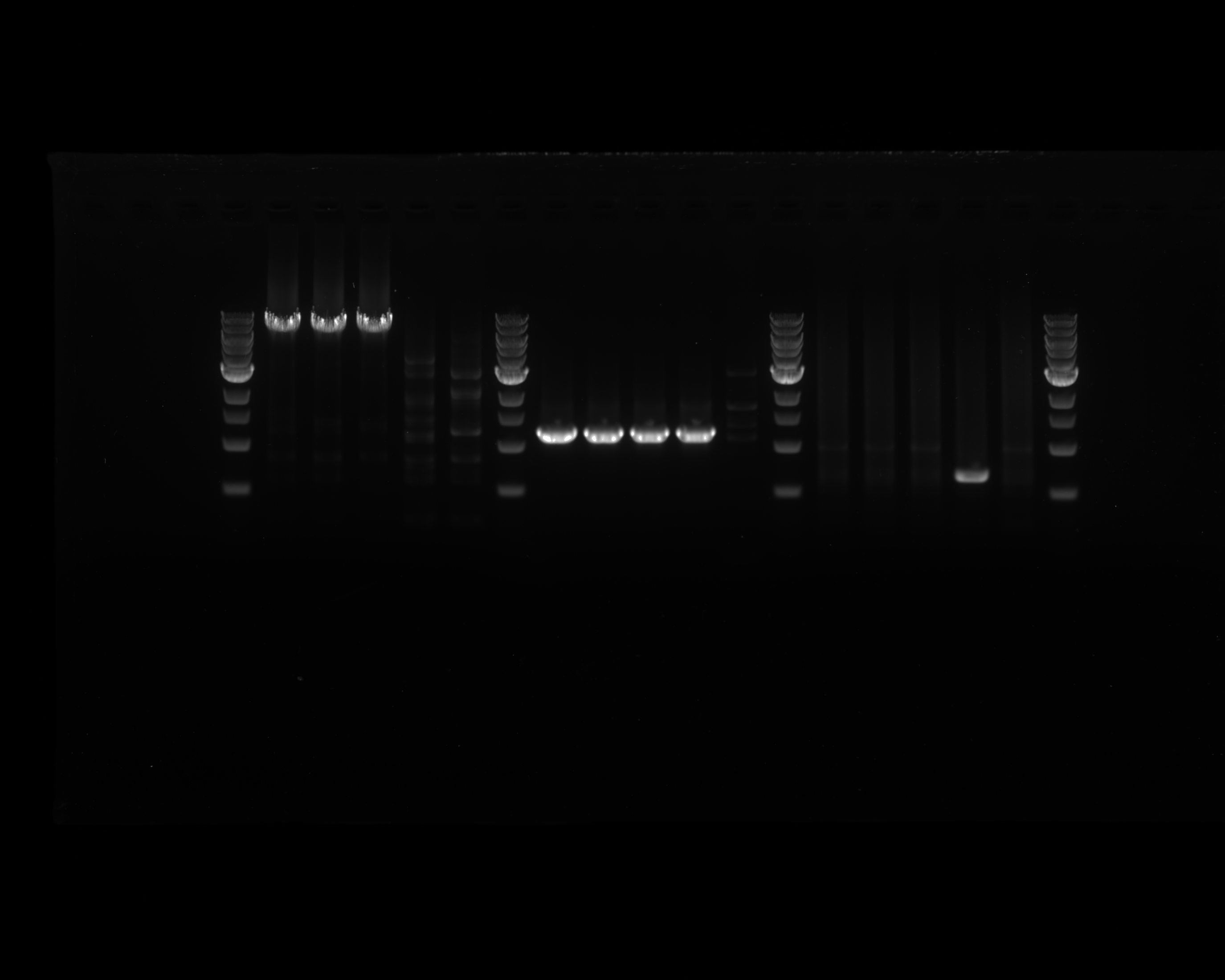

Supplement: Figure 4—figure supplement 3—source data 1. [file elife-95856-fig4-figsupp3-data1.zip › Supplementary Figure 8 - Source Data 1/Mice_F2_187sto189s.tif]

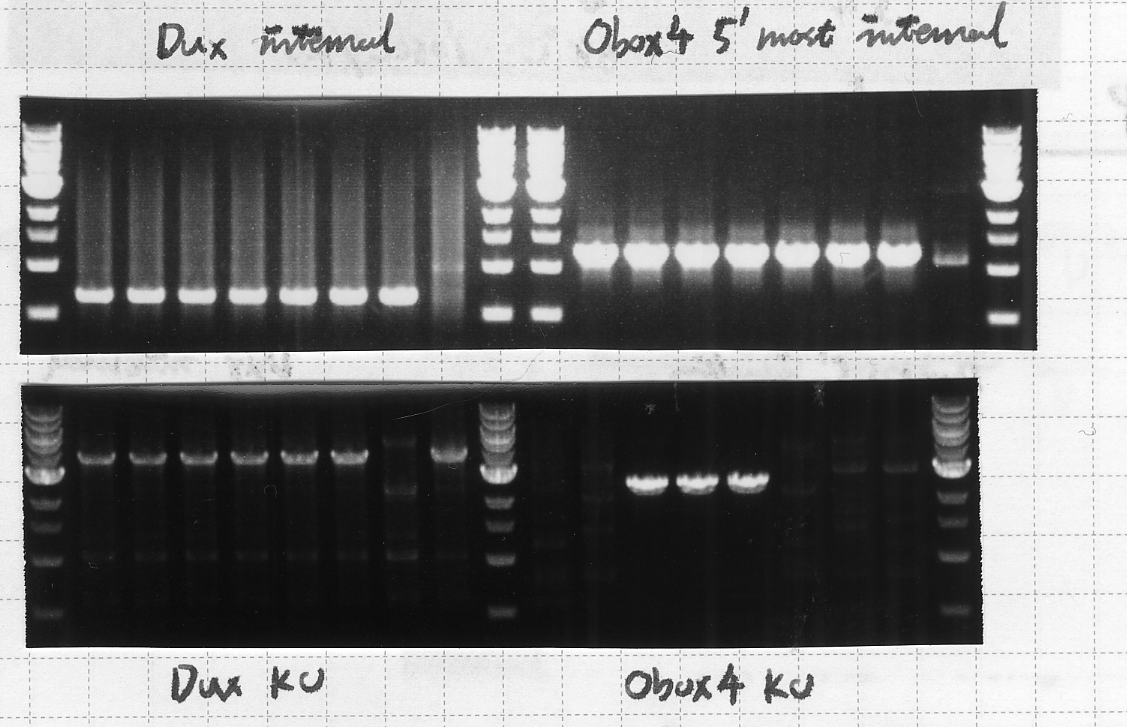

Supplement: Figure 4—figure supplement 3—source data 1. [file elife-95856-fig4-figsupp3-data1.zip › Supplementary Figure 8 - Source Data 1/Mice_F2_190sto195s.tif]

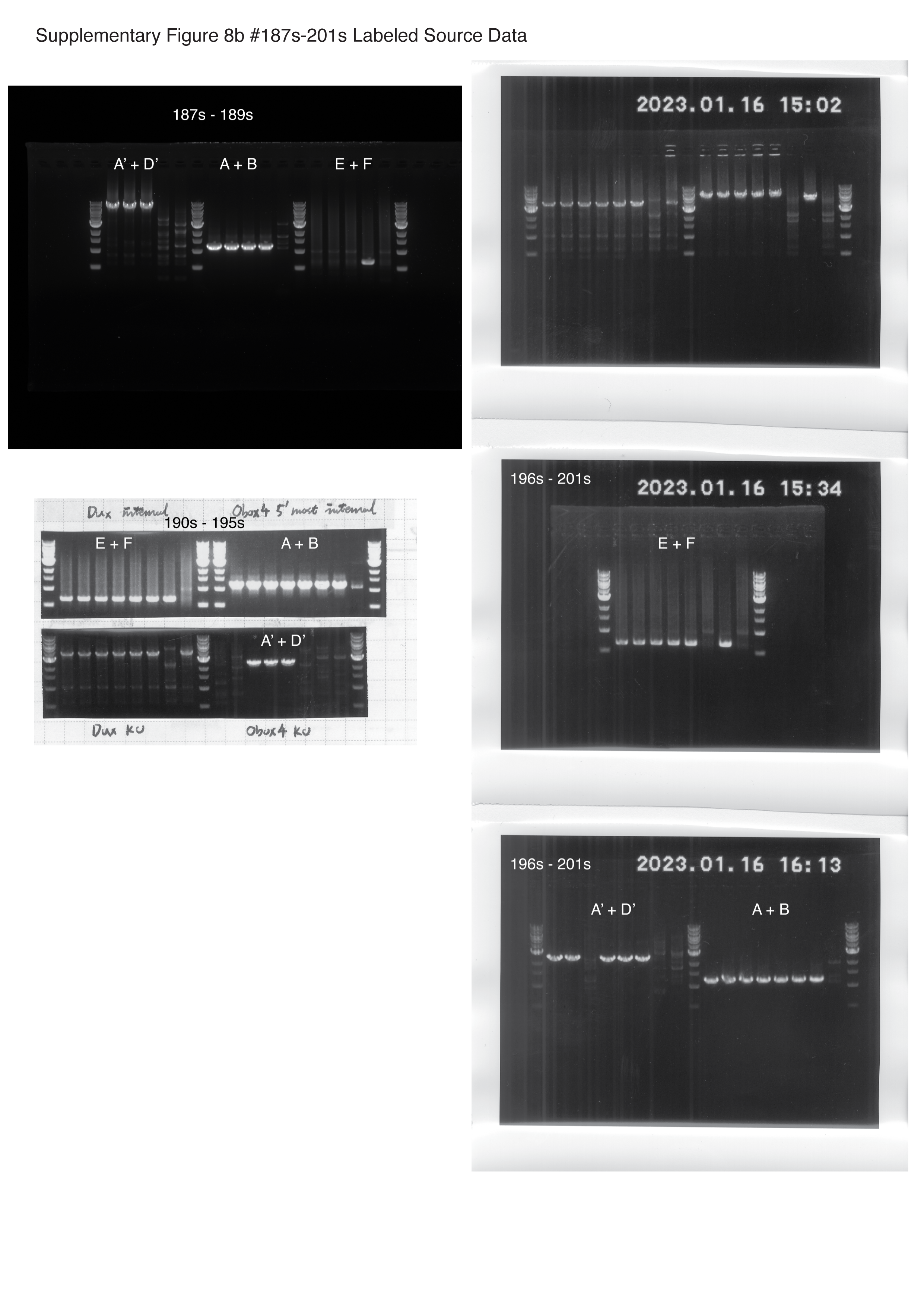

Supplement: Figure 4—figure supplement 3—source data 1. [file elife-95856-fig4-figsupp3-data1.zip › Supplementary Figure 8 - Source Data 1/Supplementary Figure 8b #187s-201s - Labeled Source Data.png]

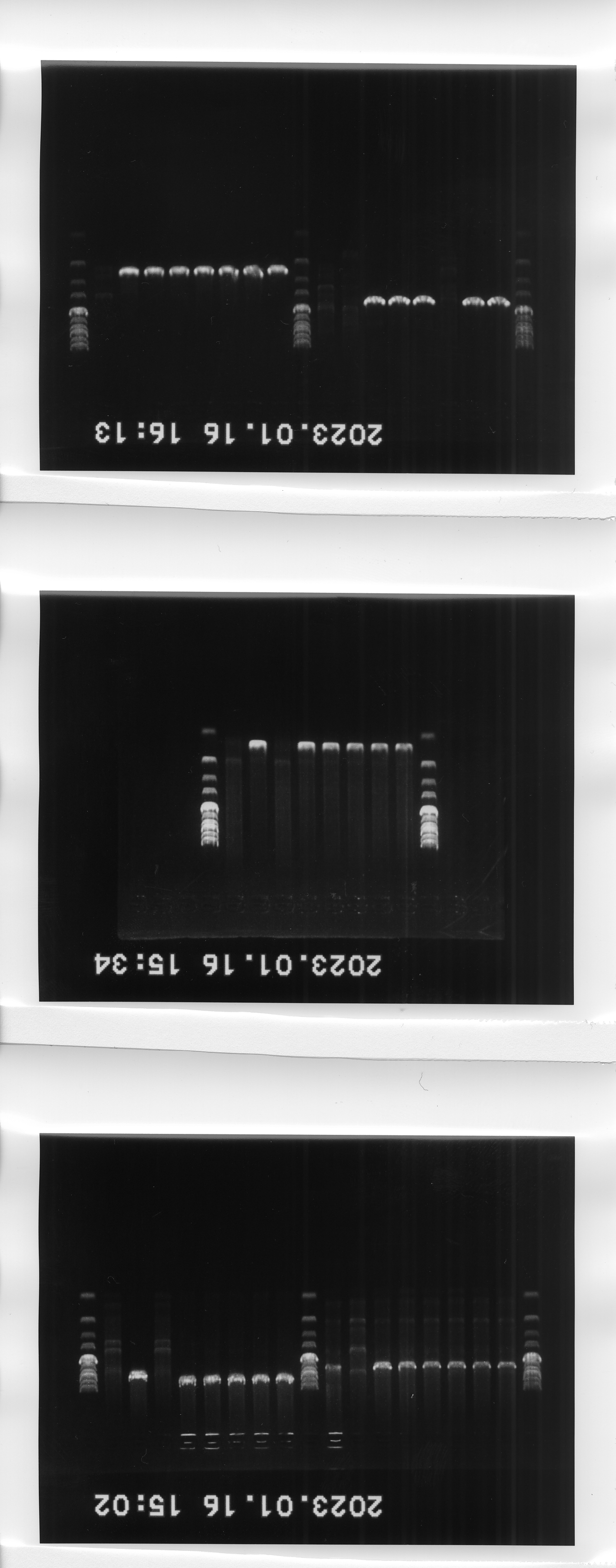

Supplement: Figure 4—figure supplement 3—source data 1. [file elife-95856-fig4-figsupp3-data1.zip › Supplementary Figure 8 - Source Data 1/Mice_F2_196sto201s.tif]

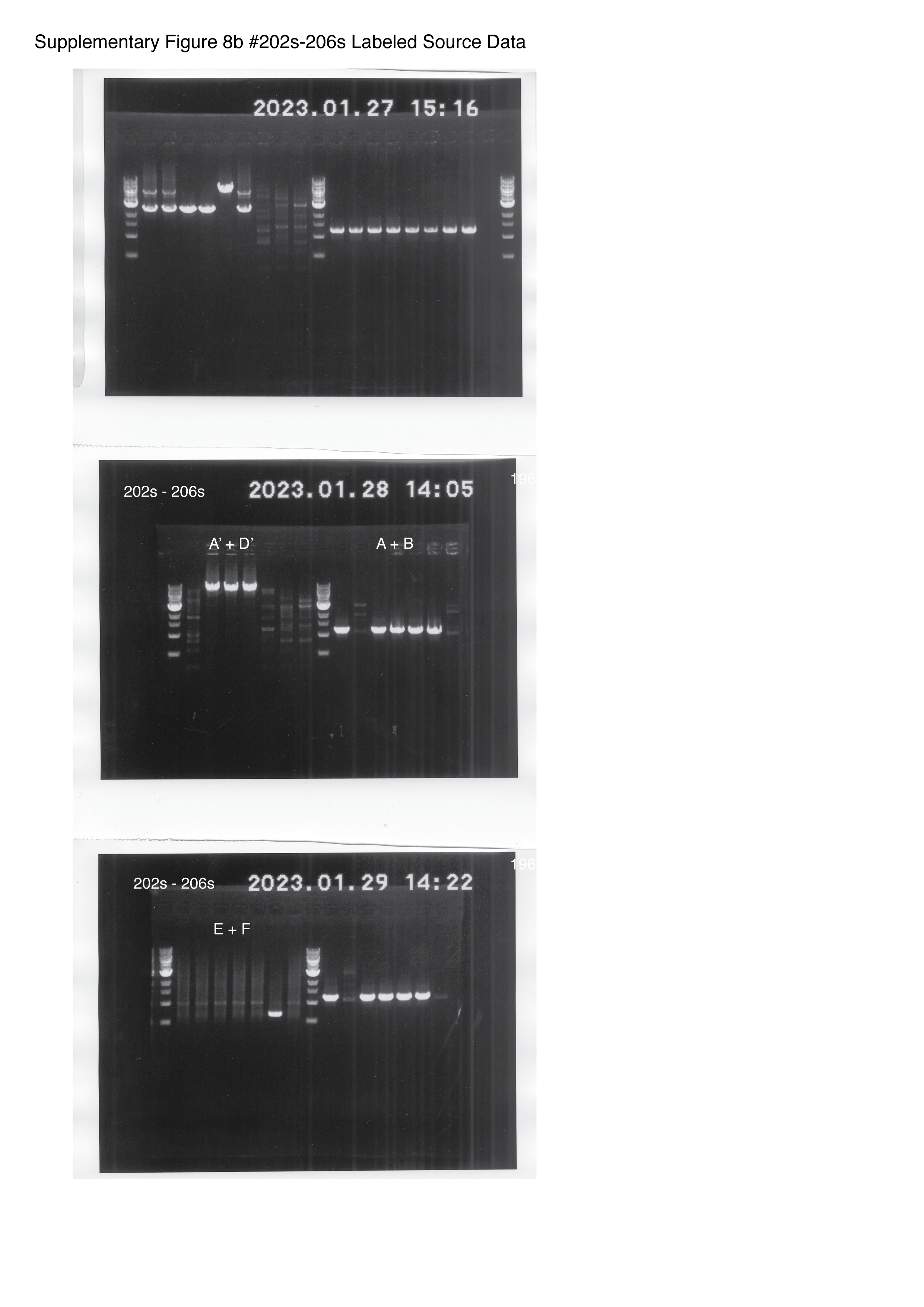

Supplement: Figure 4—figure supplement 3—source data 2. [file elife-95856-fig4-figsupp3-data2.zip › Supplementary Figure 8 - Source Data 2/Supplementary Figure 8b #202s-206s - Labeled Source Data.png]

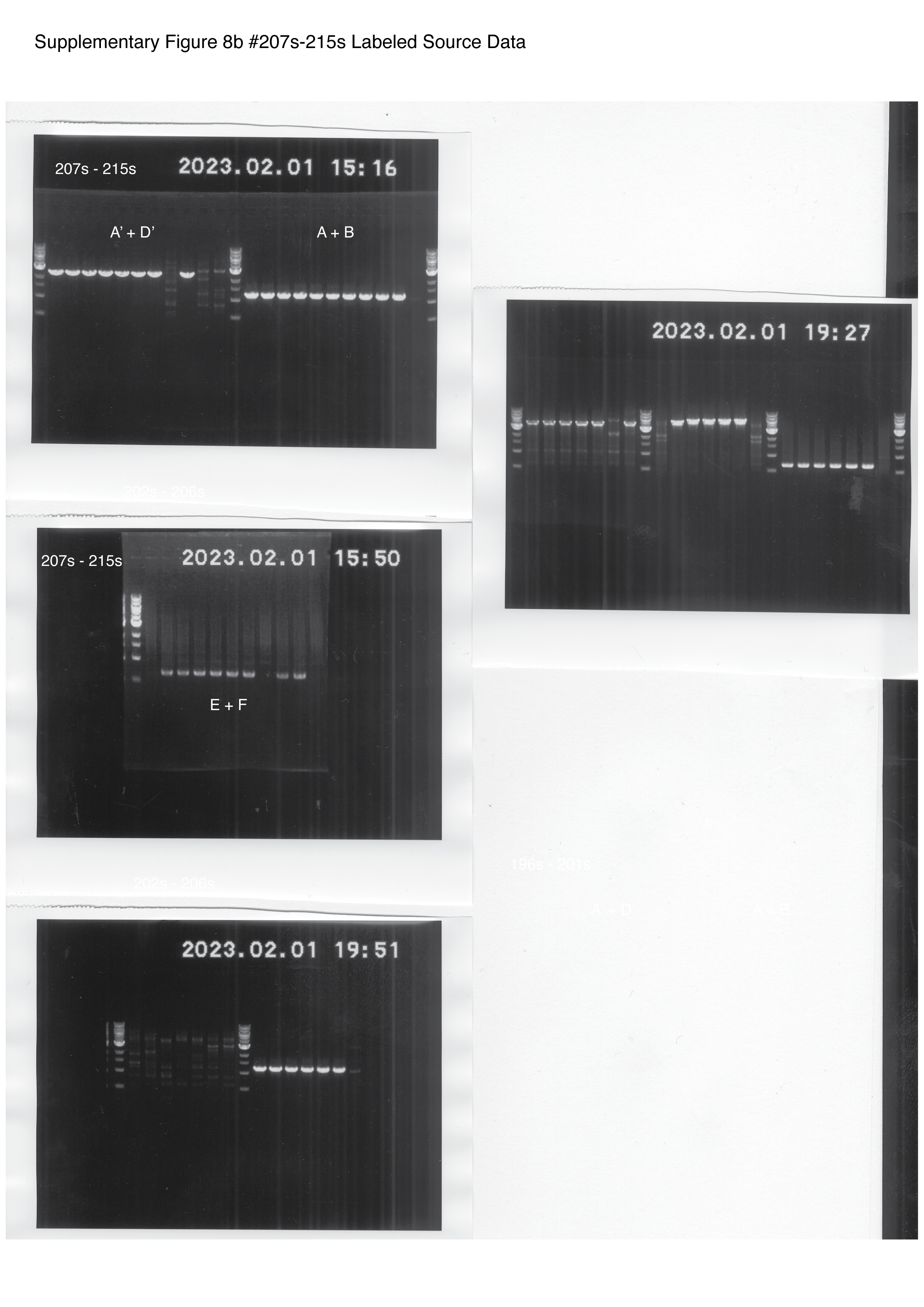

Supplement: Figure 4—figure supplement 3—source data 3. [file elife-95856-fig4-figsupp3-data3.zip › Supplementary Figure 8 - Source Data 3/Supplementary Figure 8b #207s-215s - Labeled Source Data.png]

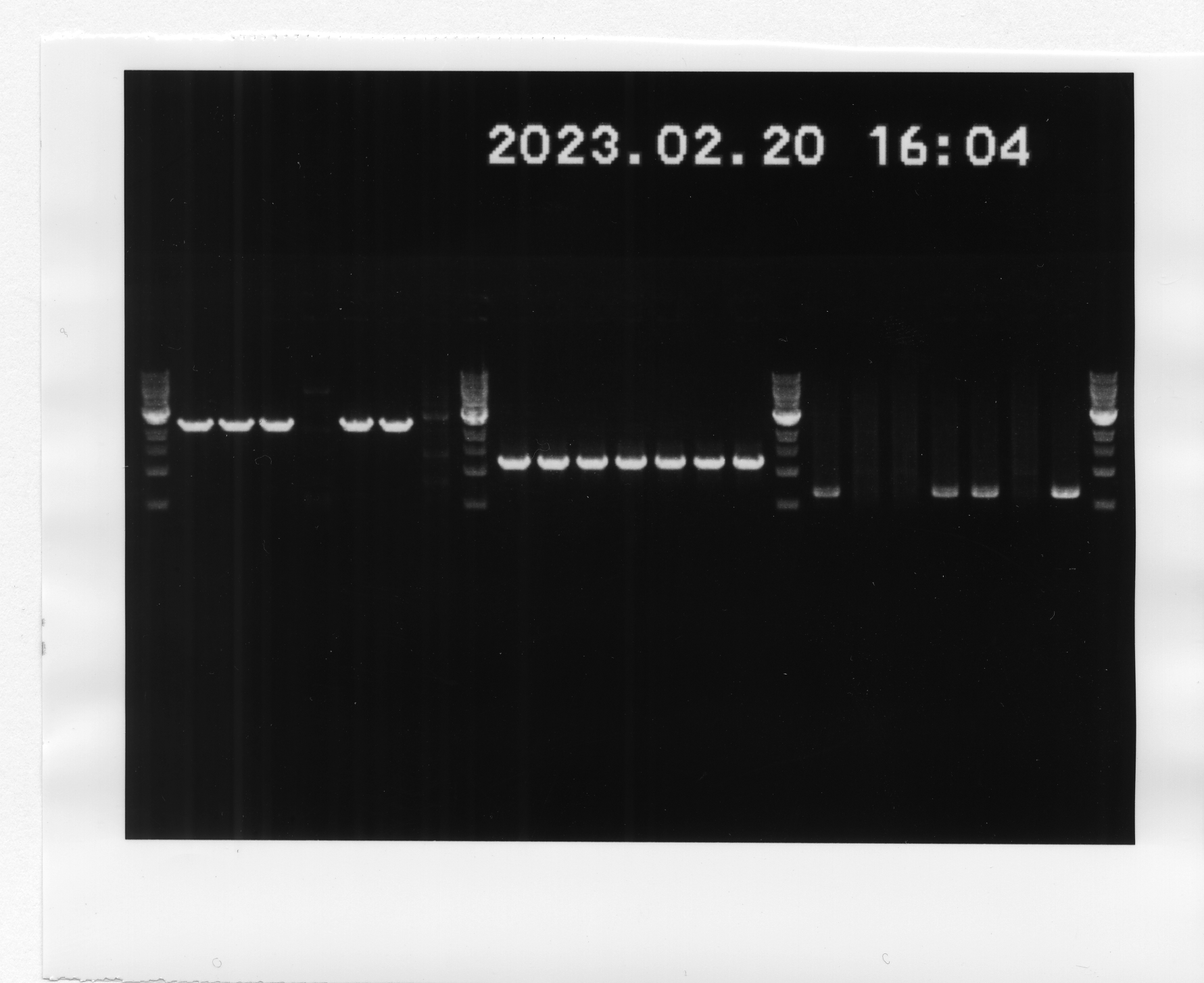

Supplement: Figure 4—figure supplement 3—source data 4. [file elife-95856-fig4-figsupp3-data4.zip › Supplementary Figure 8 - Source Data 4/Mice_F2_223sto228s.tif]

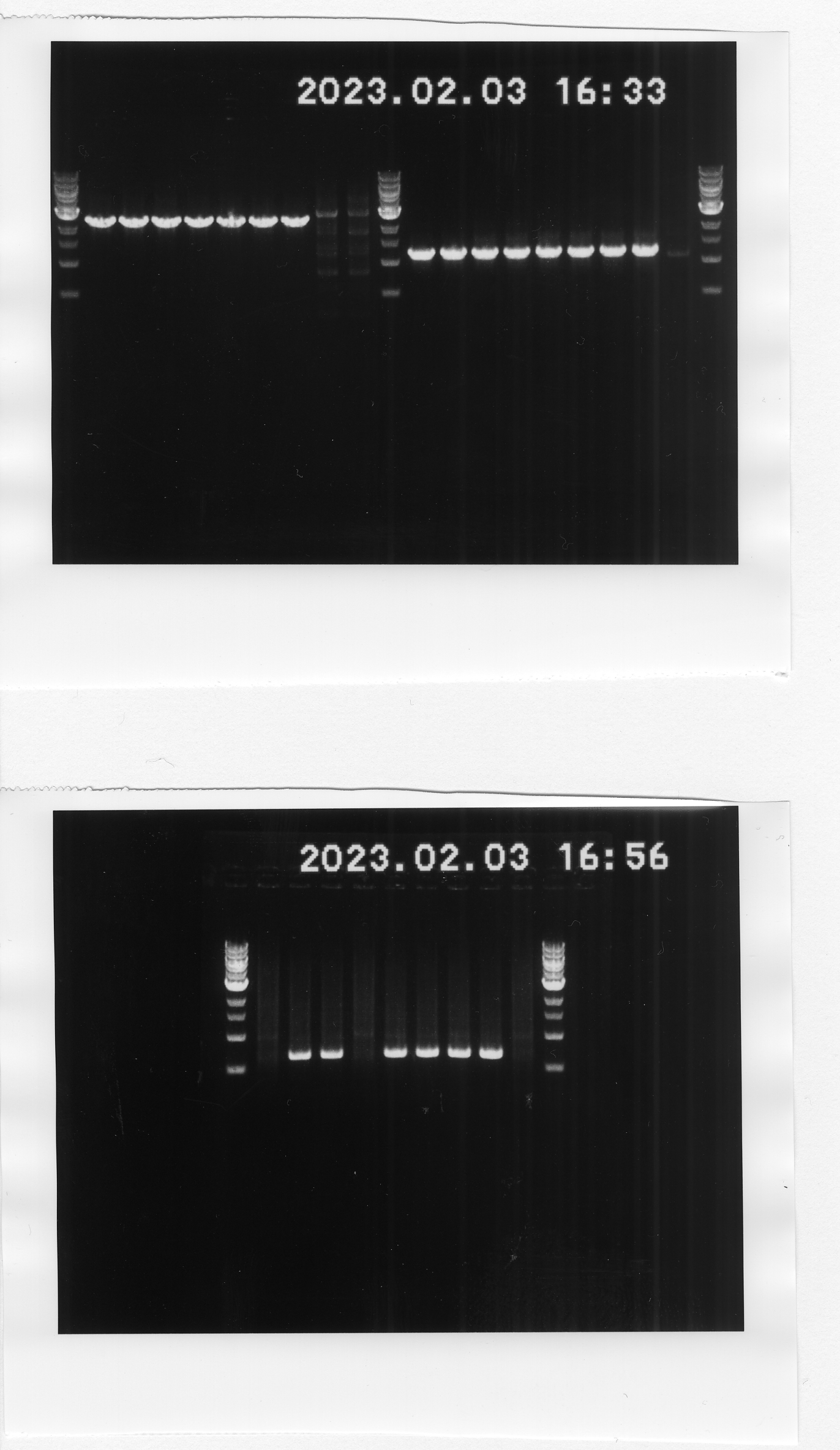

Supplement: Figure 4—figure supplement 3—source data 4. [file elife-95856-fig4-figsupp3-data4.zip › Supplementary Figure 8 - Source Data 4/Mice_F2_216sto222s.tif]

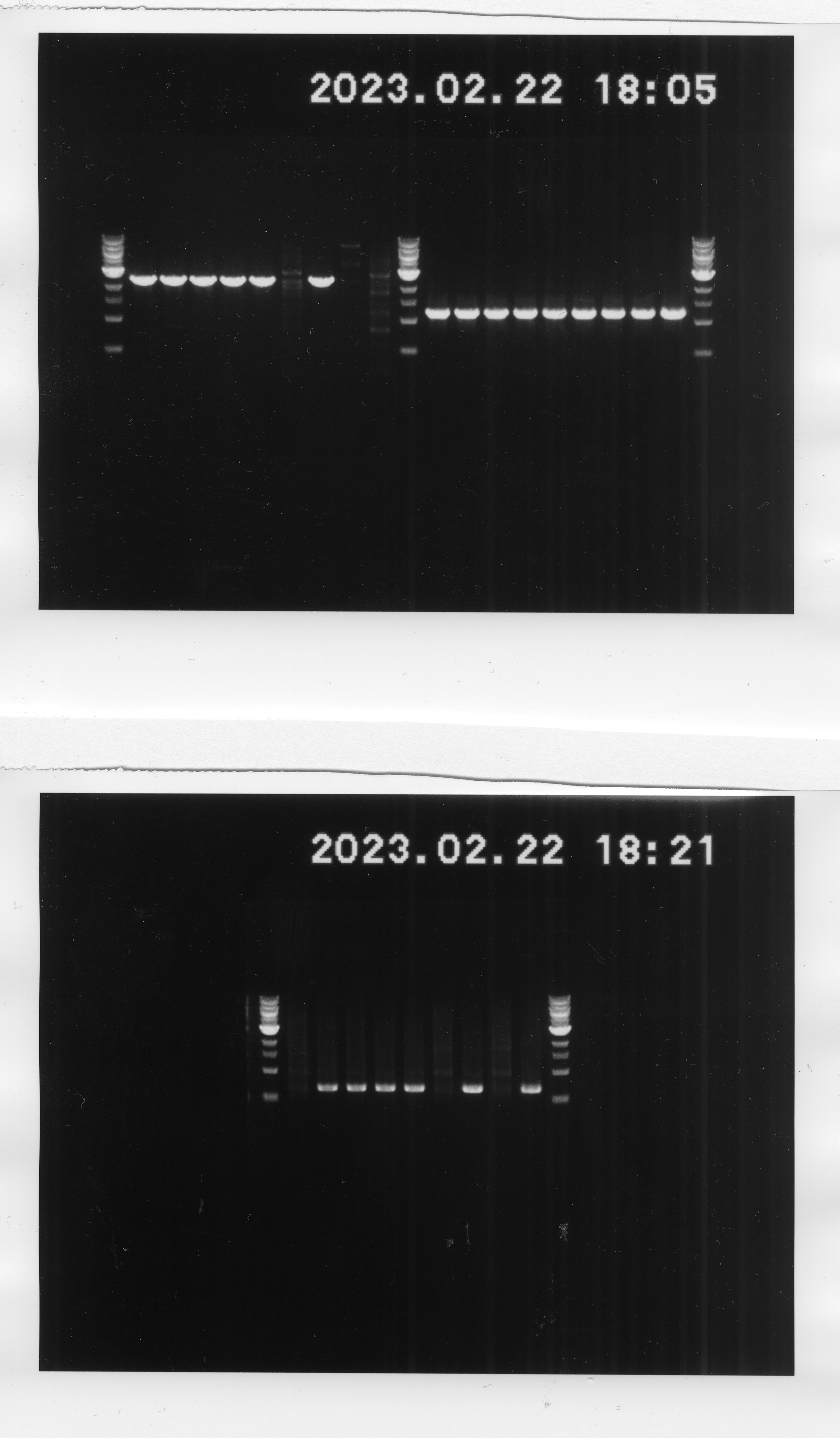

Supplement: Figure 4—figure supplement 3—source data 4. [file elife-95856-fig4-figsupp3-data4.zip › Supplementary Figure 8 - Source Data 4/Mice_F2_229sto236s.tif]

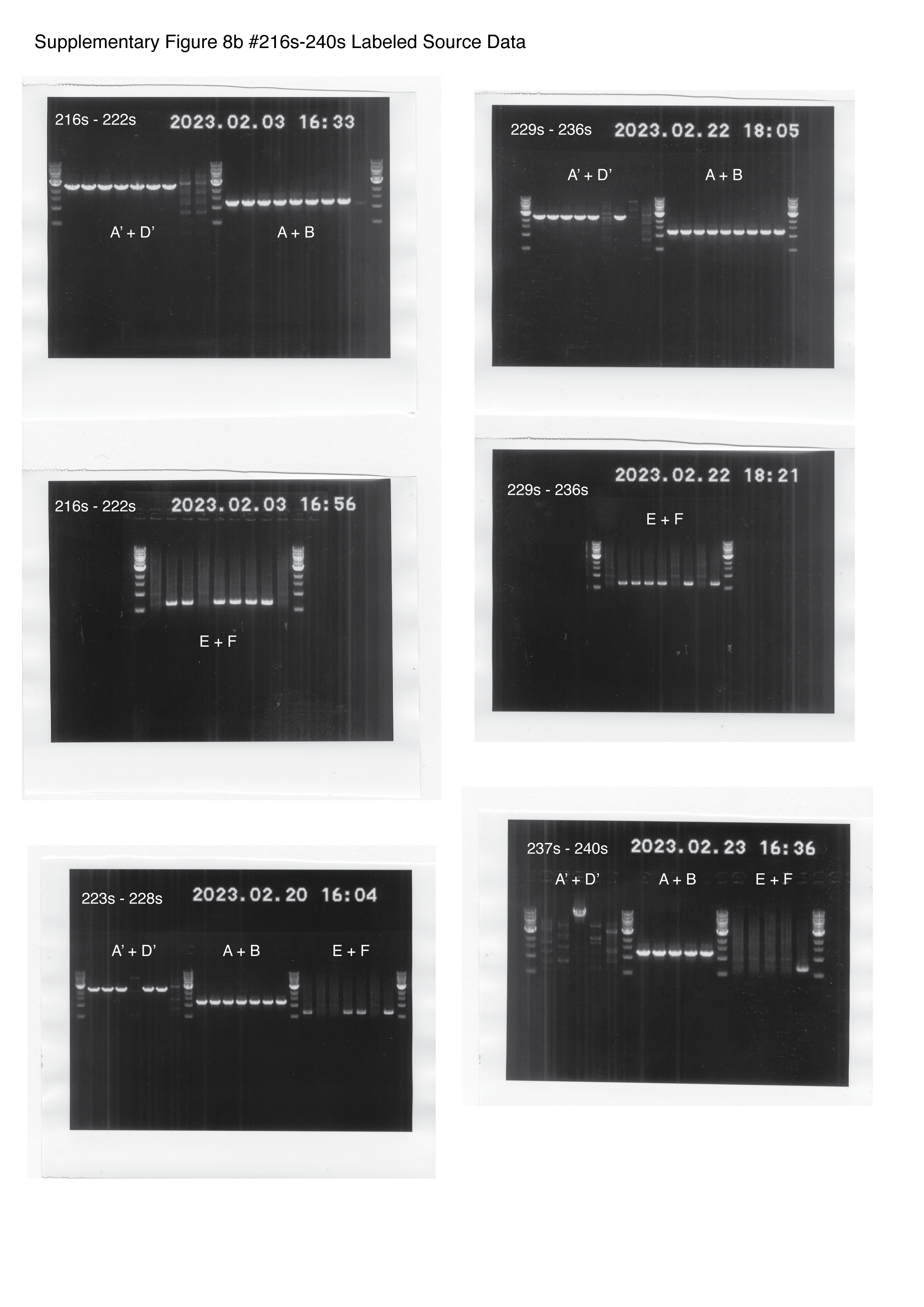

Supplement: Figure 4—figure supplement 3—source data 4. [file elife-95856-fig4-figsupp3-data4.zip › Supplementary Figure 8 - Source Data 4/Supplementary Figure 8b #216s-240s - Labeled Source Data.png]

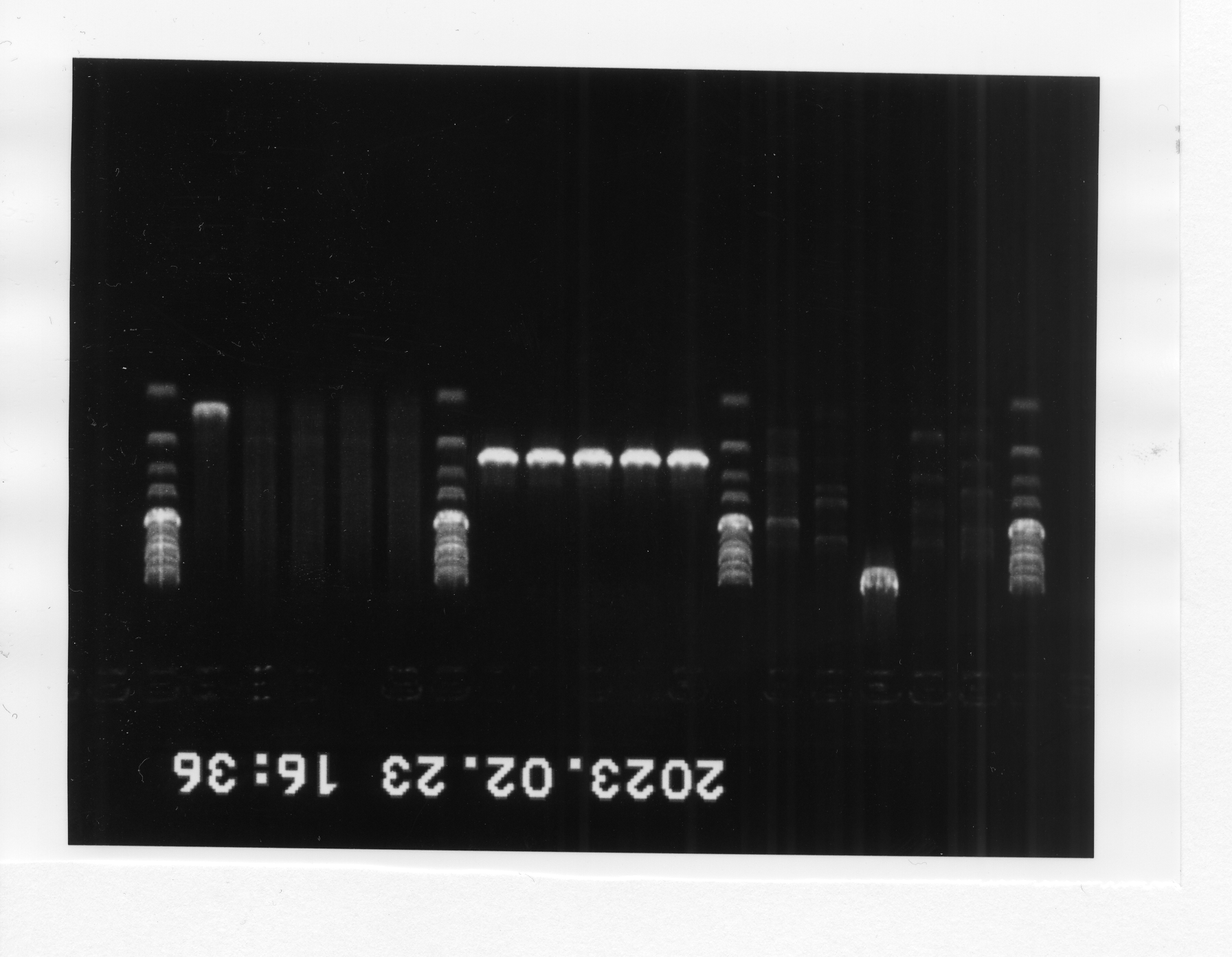

Supplement: Figure 4—figure supplement 3—source data 4. [file elife-95856-fig4-figsupp3-data4.zip › Supplementary Figure 8 - Source Data 4/Mice_F2_237sto240s.tif]

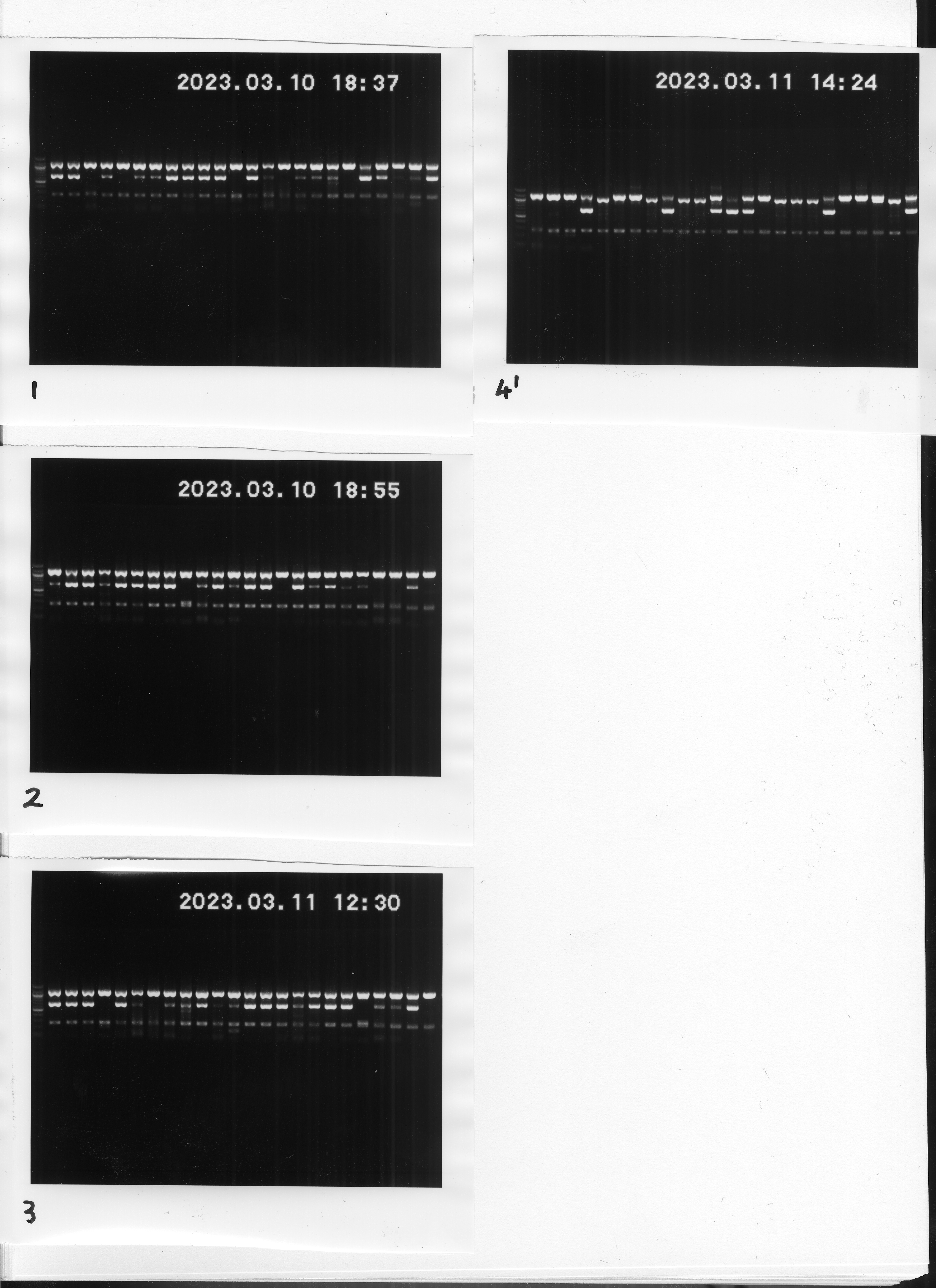

Supplement: Figure 4—figure supplement 4—source data 1. [file elife-95856-fig4-figsupp4-data1.zip › Supplementary Figure 9 - Source Data 1/Embryo_monitor.tif]

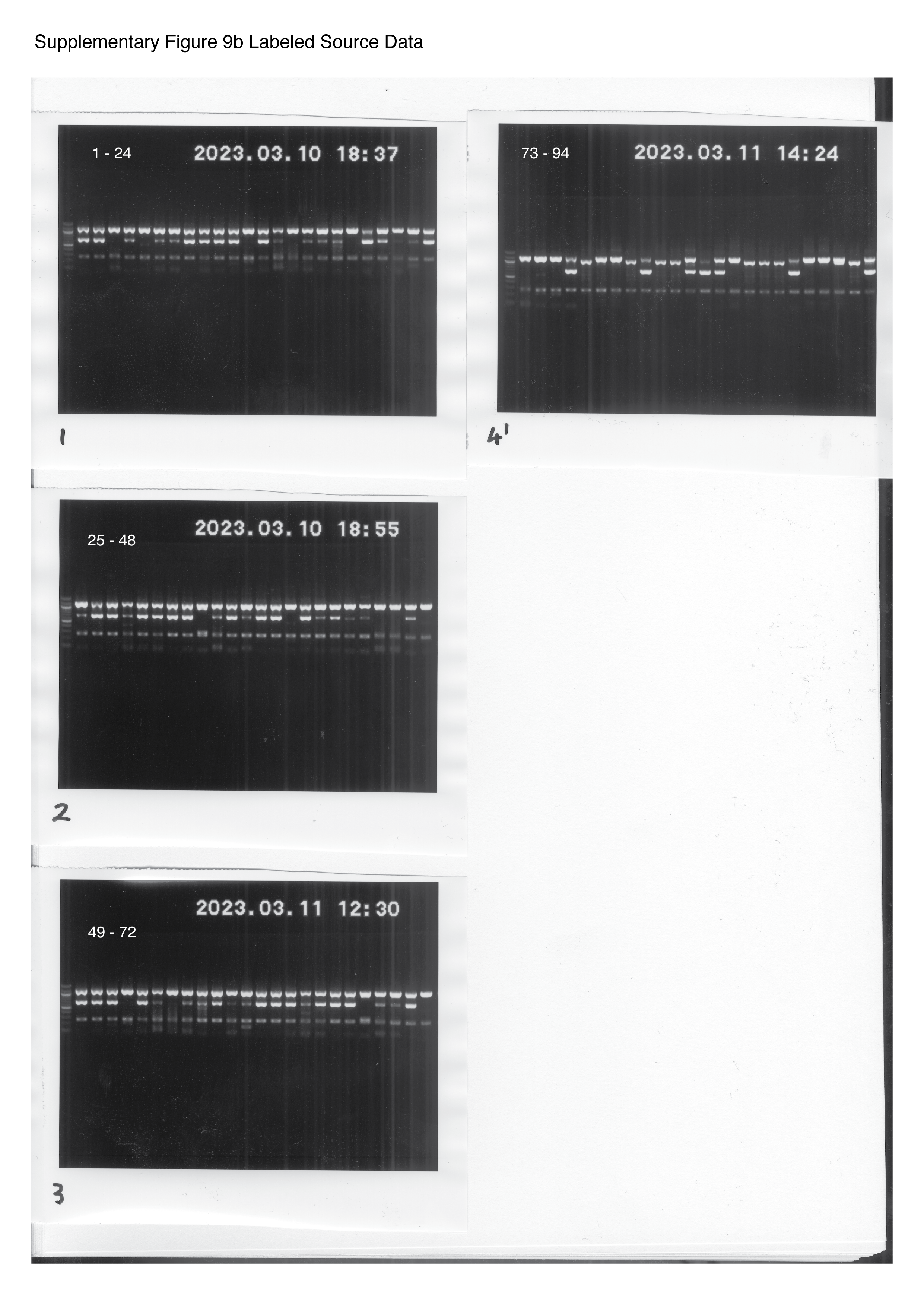

Supplement: Figure 4—figure supplement 4—source data 1. [file elife-95856-fig4-figsupp4-data1.zip › Supplementary Figure 9 - Source Data 1/Supplementary Figure 9b - Labeled Source Data.png]

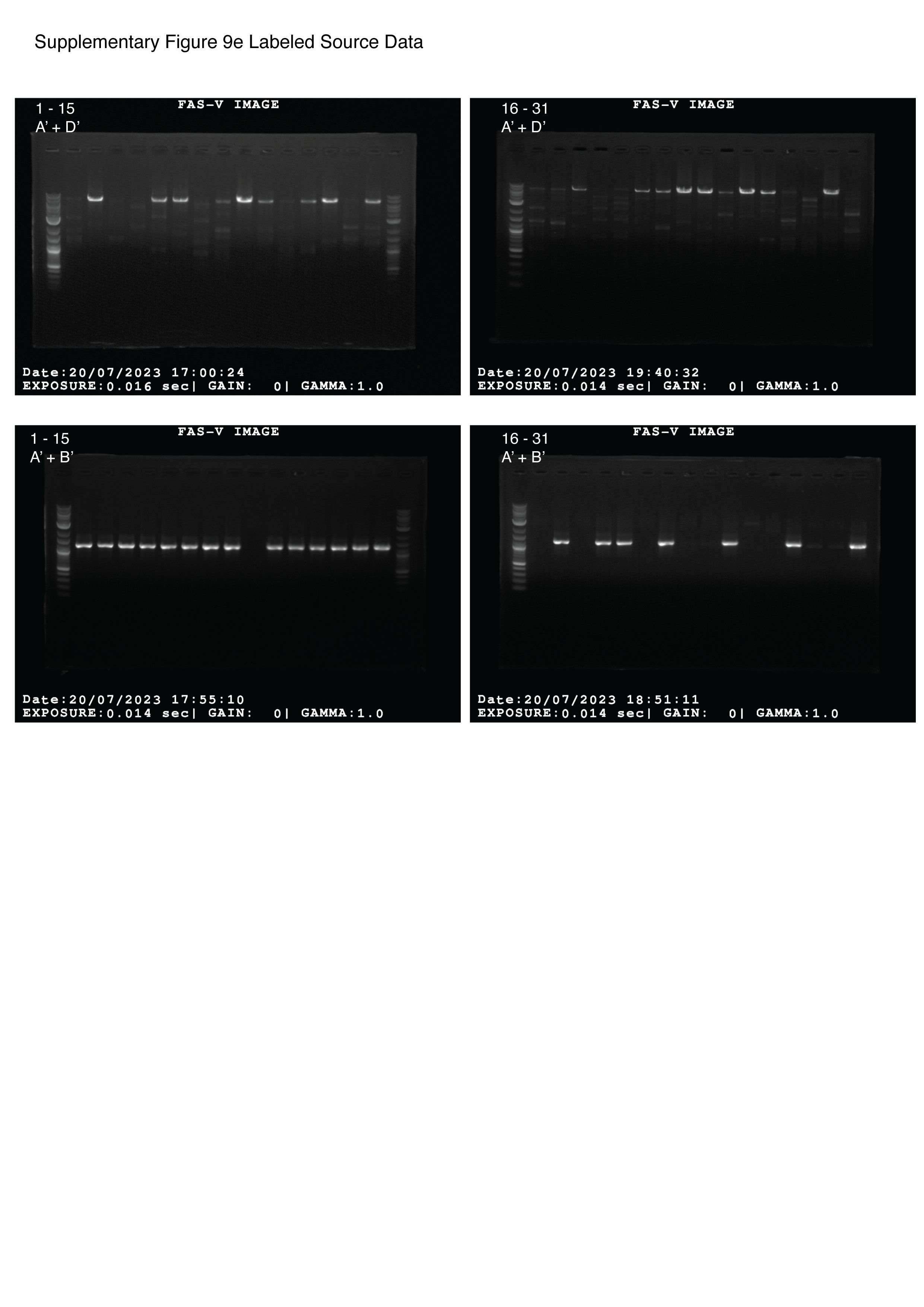

Supplement: Figure 4—figure supplement 4—source data 2. [file elife-95856-fig4-figsupp4-data2.zip › Supplementary Figure 9 - Source Data 2/Supplementary Figure 9e - Labeled Source Data.png]
